# Crack Detection in Fibre Reinforced Plastic Structures Using Embedded Fibre Bragg Grating Sensors: Theory, Model Development and Experimental Validation

**DOI:** 10.1371/journal.pone.0141495

**Published:** 2015-10-29

**Authors:** G. F. Pereira, L. P. Mikkelsen, M. McGugan

**Affiliations:** Technical University of Denmark, Department of Wind Energy, Roskilde, Denmark; Washington State University, UNITED STATES

## Abstract

In a fibre-reinforced polymer (FRP) structure designed using the emerging *damage tolerance* and *structural health monitoring* philosophy, sensors and models that describe crack propagation will enable a structure to operate despite the presence of damage by fully exploiting the material’s mechanical properties. When applying this concept to different structures, sensor systems and damage types, a combination of damage mechanics, monitoring technology, and modelling is required. The primary objective of this article is to demonstrate such a combination. This article is divided in three main topics: the damage mechanism (delamination of FRP), the structural health monitoring technology (fibre Bragg gratings to detect delamination), and the finite element method model of the structure that incorporates these concepts into a final and integrated damage-monitoring concept. A novel method for assessing a crack growth/damage event in fibre-reinforced polymer or structural adhesive-bonded structures using embedded fibre Bragg grating (FBG) sensors is presented by combining conventional measured parameters, such as wavelength shift, with parameters associated with measurement errors, typically ignored by the end-user. Conjointly, a novel model for sensor output prediction (virtual sensor) was developed using this FBG sensor crack monitoring concept and implemented in a finite element method code. The monitoring method was demonstrated and validated using glass fibre double cantilever beam specimens instrumented with an array of FBG sensors embedded in the material and tested using an experimental fracture procedure. The digital image correlation technique was used to validate the model prediction by correlating the specific sensor response caused by the crack with the developed model.

## Introduction

### Damage Tolerant Design and Structural Health Monitoring in Fibre-Reinforced Polymer Material Structures

Fibre-reinforced polymer materials (FRP, often referred to as composite materials) have been extensively used in aerospace, automotive, naval, wind energy and civil engineering applications, mostly due to their high stiffness/weight ratio. A fibre-reinforced polymer composite material consists of two distinct macroscopic phases: a stiff phase (generally glass or carbon) and a polymer matrix. One of the advantages of FRP material is their ability to be tailored for a specific application; this enables an enhancement and a high level of customisation of their mechanical properties. Thus, in a FRP structure, it is possible to align the reinforcement in the directions where higher stiffness is required, which makes the structure lighter compared with the structure of a conventional material [1].

Currently, the higher demand for more cost-effective, light-weight FRP structures is pushing advances in material technology and design philosophy. In this way, the design philosophy of FRP structures that is based on conservative analysis methods, with large safety factors, underestimation of the material properties, and considering only the linear behaviour of the materials, is becoming obsolete. A shift in the design philosophy has been discussed by several authors [2, 3], where the concept of damage tolerance is suggested as an energy concept based on a particular combination of structural design, loading environment, and material characteristics, which will enable the structure to operate despite the presence of damage. However, a standalone damage tolerance approach will not be achieved until all physical phenomena present in the FRP field are fully understood. The solution starts by accepting the presence of damage and its unpredictability, tracking this damage using a structural health monitoring approach, where sensors integrated during manufacturing will provide information about the presence of damage in an accurate way, its location, the type of damage and the remaining operating life of the structure.

### Article Objectives

The main objective of this article is to provide a better understanding of the different fields that need to be addressed to design a structure using a *damage tolerance* and *structural health monitoring* philosophy, as well as a methodology that can follow this concept to different structures, sensor systems and damage types.

To achieve this goal, it is necessary to explore three different fields in more detail; thus, the following key concepts are linked and fully described.

- Damage mechanism: delamination in FRP as a damage tolerance property of the structure. Fracture mechanism and stress distributions along the crack/damage area.
- Structural health monitoring technology: embedded fibre Bragg gratings to detect and track cracks/delamination in FRP structures. FBG working principle.
- Finite element method (FEM) model of the structure: incorporation of the damage mechanism with the structural health monitoring technology to a final and integrated *damage-monitoring concept*. Virtual FBG: FEM sensor output model for FRP delamination.

## Delamination as a Damage Tolerant Mechanism

**Fig 1:**
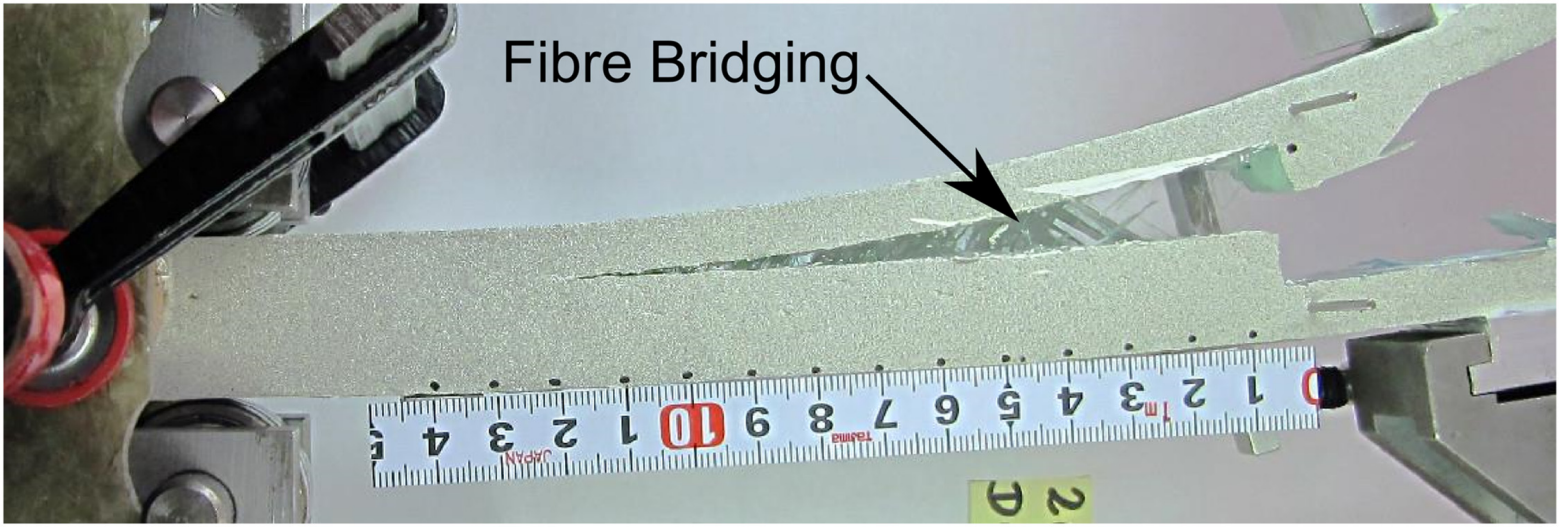
Fibre bridging phenomenon during delamination in a glass fibre specimen.

Interface fracture resulting from crack growth along interfaces in laminated structures is called delamination, and it can be considered as the most widespread cause of life reduction and one of the most important failure mechanisms in FRP structures. Delamination can be analysed through fracture mechanics; thus, damage tolerance implies that the crack growth is stable and that the energy required for unstable crack growth (catastrophic event) is higher than the energy level required to initiate the crack. This damage tolerant mechanism can be defined as a crack bridging phenomenon, i.e., the delamination is accompanied by the formation of a fracture process zone, in which intact fibres connect the crack faces behind the crack tip, as shown in Fig 1, which increases the energy required for a crack to grow.

This large-scale crack bridging zone cannot be addressed by linear elastic fracture mechanics (LEFM). Rather, a cohesive model can be used to describe the fracture process zone [4]. The cohesive law *σ*_*n*_(*δ*_*n*_) can be briefly described as a normal traction, *σ*_*n*_, as function of the normal opening, *δ*_*n*_, in the active cohesive zone [5].

### Stress Distribution in the Crack/Damage Area

To successfully detect the growth of a crack in an FRP material, the measurement technique should track specific fracture features that only occur in the vicinity of a crack. Thus, the stress distribution around the crack tip in an FRP specimen was analysed. This allowed the different measured parameters to be linked with all the different fracture features.

**Fig 2:**
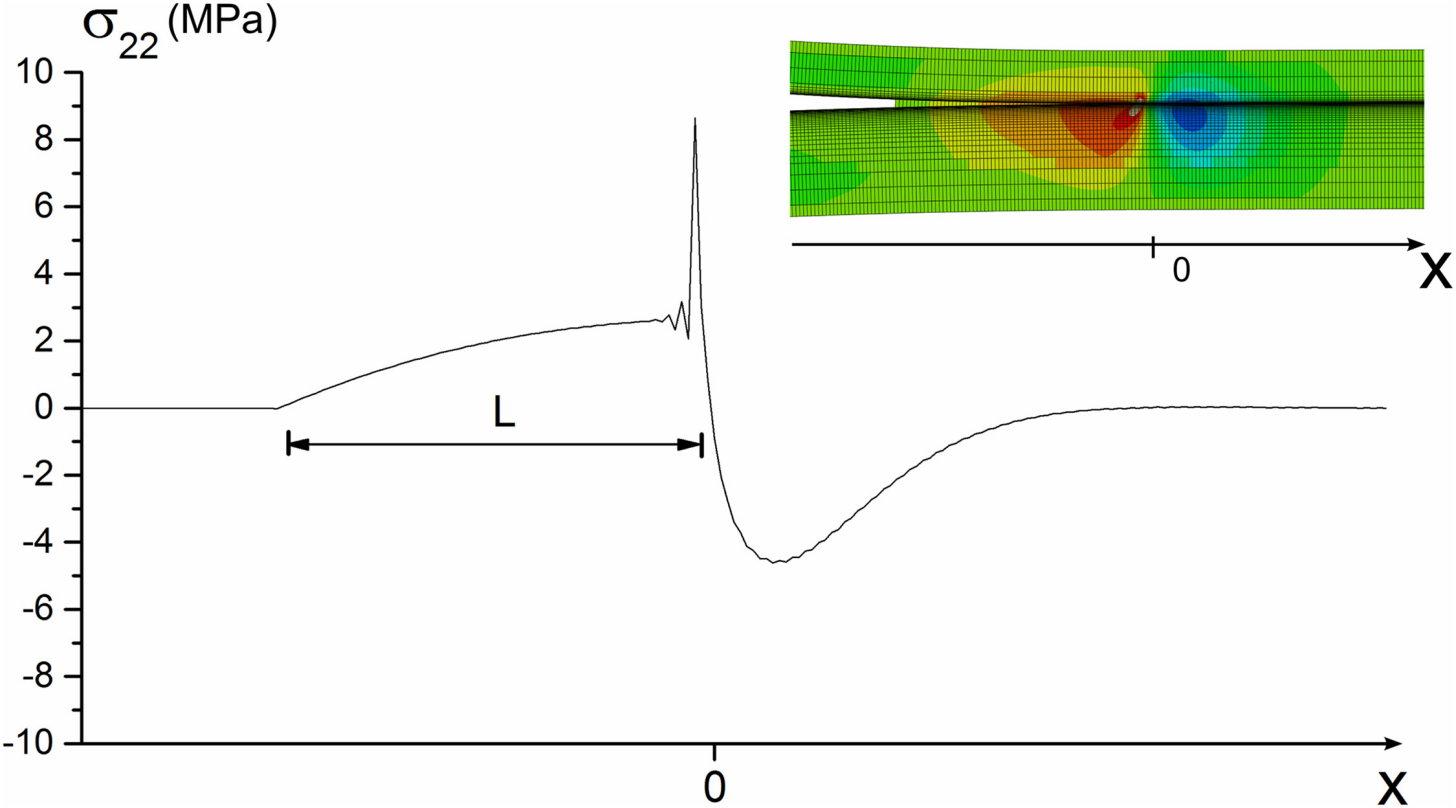
Finite element method simulation: stress *σ*_22_ distribution at the fracture process zone (FPZ) for Mode I fracture.

**Fig 3:**
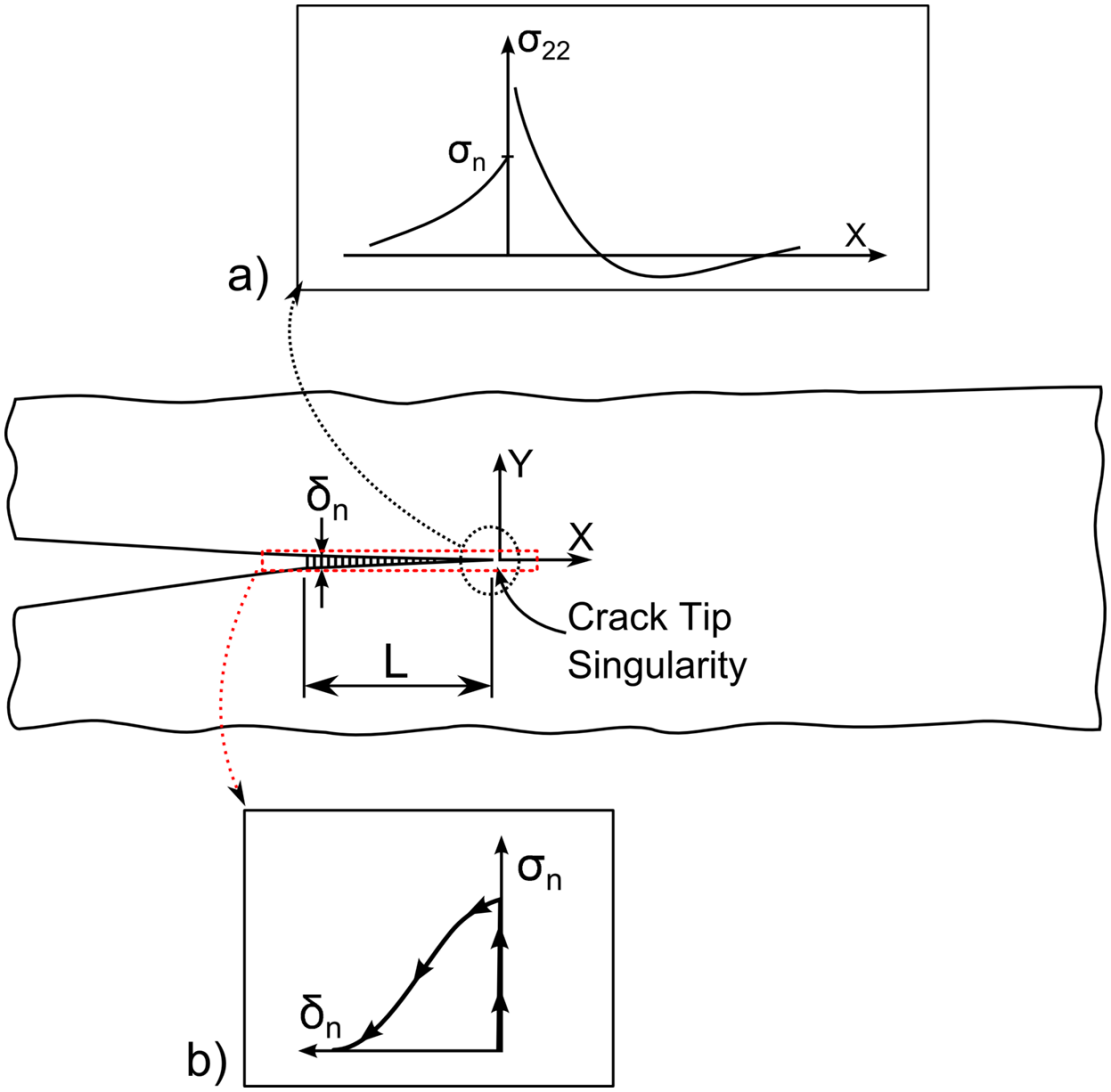
Illustration of bridging zone stress distribution. (a) Crack tip singular stress field and (b) schematic of a bridging law: relationship between the normal stress, *σ*_*n*_, and separation, *δ*_*n*_, across the FPZ.

The fracture process zone (FPZ) is a region near the crack tip where the material strength is locally reduced. The stress distribution in the FPZ can be divided into two distinct contributions: the crack tip singularity at the front of the FPZ and the crack bridging at the FPZ wake. Near the crack tip, the singularity dominated the zone (K-dominant). The stress field closely approached the singular stress field of LEFM, indicating that the stress tends to infinity, creating a high stress gradient area, as shown in Figs 2 and 3a).

Considering the crack tip where the material is developing damage at *x* = 0, in the fracture process zone given by −*L* < *x* < 0, the material is damaged, and its ability to transfer stress is decreased, as described by the cohesive law. This FPZ is characterised by a positive stress zone, as shown in Figs 2 and 3b), which is balanced by a compression zone ahead of the crack tip (*x* > 0). The size of the compression zone will depend on the cohesive law and the material parameters [4].

## Structural Health Monitoring

Accepting damage and incorporating it as part of the design process will require full control over the structural integrity. A structural health monitoring system’s main purpose is to provide information about the presence of damage in an accurate way, its location with good resolution, and the prognosis for the remaining life of the structure. Some techniques have already been implemented to detect cracks and monitor their growth, such as acoustic emission [6], where ultrasonic stress waves generated by crack growth are detected; vibration [7], by measuring the change in the specific damping capacity; modal analysis [8], by monitoring the natural frequencies and mode shapes; piezoelectric actuators/sensors; and wavelet analysis [9] based on the energy variation in the structural dynamics. However, applying these techniques in operational structures presents some difficulties due to technical limitations, the need for manual inspections performed by qualified operators, expensive hardware, and so forth.

### Fibre Optic Sensors as Structural Health Monitoring Technology

Fibre optic sensors, such fibre Bragg gratings (FBG), have the ability to perform damage/failure monitoring during the operation of a structure without compromising its performance and structural resistance. The small size of an FBG, a diameter of 125 *μm*, makes it virtually non-intrusive when embedded in the material. Additionally, FBG sensors have high resolution, multiple measurement points per fibre capability (multiplexing), immunity to electromagnetic fields, chemical inertness, immunity to optical power fluctuations, and long-term stability. These characteristics make embedded FBGs a very promising technology for tracking cracks in composite materials.

Knowing that an embedded FBG sensor will be under the influence of different fracture phenomena during a crack growth event, such as a crack bridging zone, where intact fibres connect the crack faces and a stress concentration zone near the crack tip that influences the stress distribution (stress gradient), being able to identify and measure these specific phenomena is the key factor for determining the presence of damage and its growth in a structure. In the next section, FBG sensor responses for three different stress/strain states that occur during crack growth are presented.

### Fibre Bragg Grating Sensor

**Fig 4:**
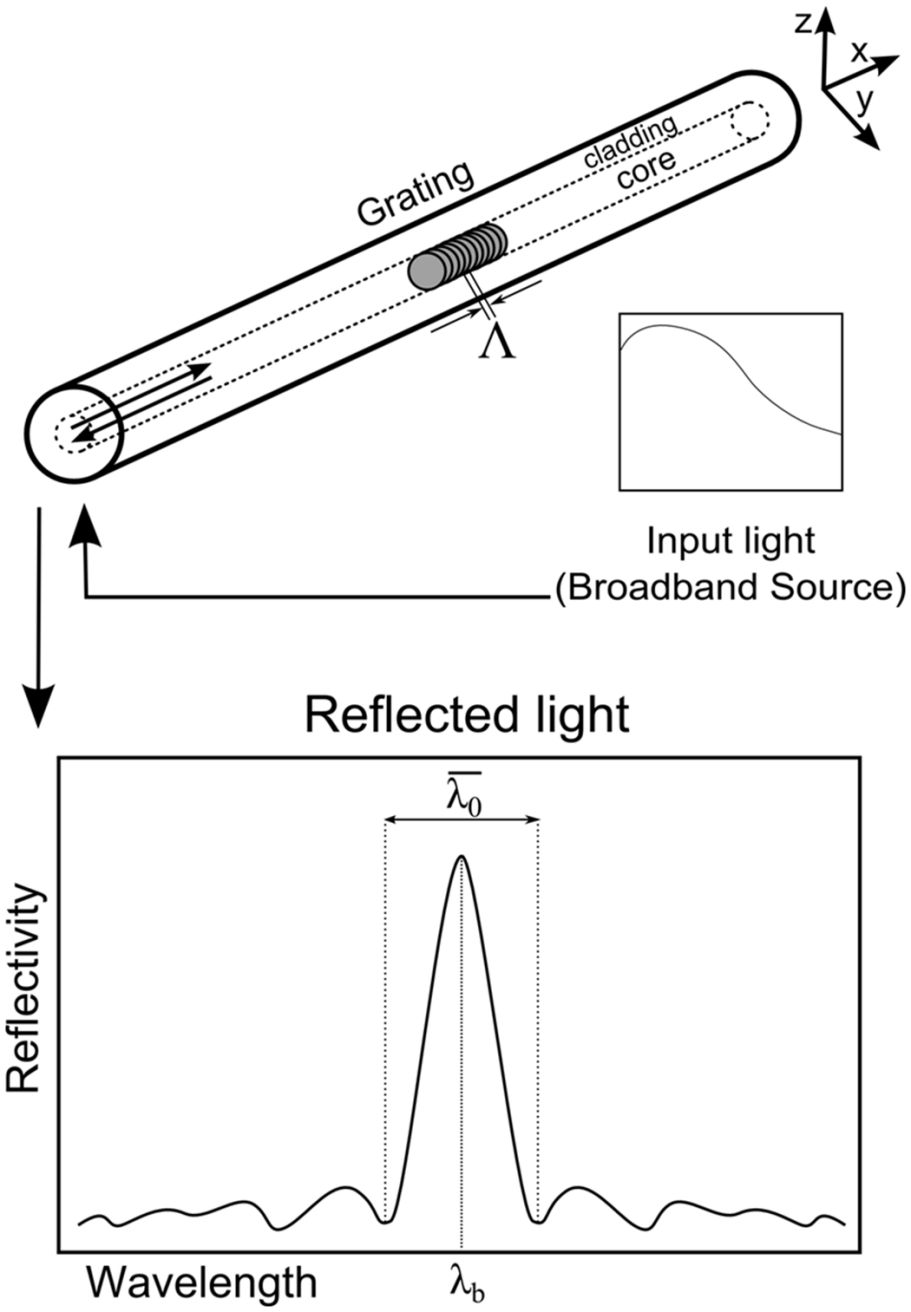
Fibre Bragg grating response in a free state.

A fibre Bragg grating is formed by a permanent periodic modulation of the refractive index along a section of an optical fibre grating by exposing the optical fibre to an interference pattern of intense ultra-violet light [10]. The photosensitivity of the silica exposed to the ultra-violet light is increased; thus, when the optical fibre is illuminated by a broadband light source, the grating diffraction properties are such that only a very narrow wavelength band is reflected back, as shown in Fig 4.

In a free state, without strain and at a constant temperature, the spectral response of a homogeneous FBG is a single peak centred at wavelength *λ*_*b*_, with a certain bandwidth $\bar{\lambda_{0}}$ (distance between the two first minima), as shown in Fig 4. The wavelength *λ*_*b*_ is described by the Bragg condition,

$$\begin{array}{c} \lambda_{b}=2n_{eff,0}\Lambda_{0} \end{array}$$ (1)

where *n*_*eff*,0_ is the mean effective refractive index at the location of the grating, the index 0 denotes unstrained conditions (initial state), and Λ_0_ is the constant nominal period of the refractive index modulation [11]. The bandwidth is given by

$$\begin{array}{c} \frac{\bar{\lambda_{0}}}{\lambda_{b}}=\frac{1}{n_{eff,0}}\sqrt{{\left(\xi \bar{\delta n_{eff,0}}\right)}^{2}+{\left(\lambda_{b}/L\right)}^{2}} \end{array}$$ (2)

where *L* is the gauge length, $\bar{\delta n_{eff,0}}$ is the mean induced change in *n*_*eff*,0_, and *ξ* is the amplitude of the induced index change [12]. An external load or temperature variation will change the effective index of refraction and/or the period of modulation; this will create a shift of the wavelength reflected peak from its original value.

#### Response to Uniform Axial Strain

In the following sections, the temperature is assumed to be constant and to have no effect on the sensor response.

**Fig 5:**
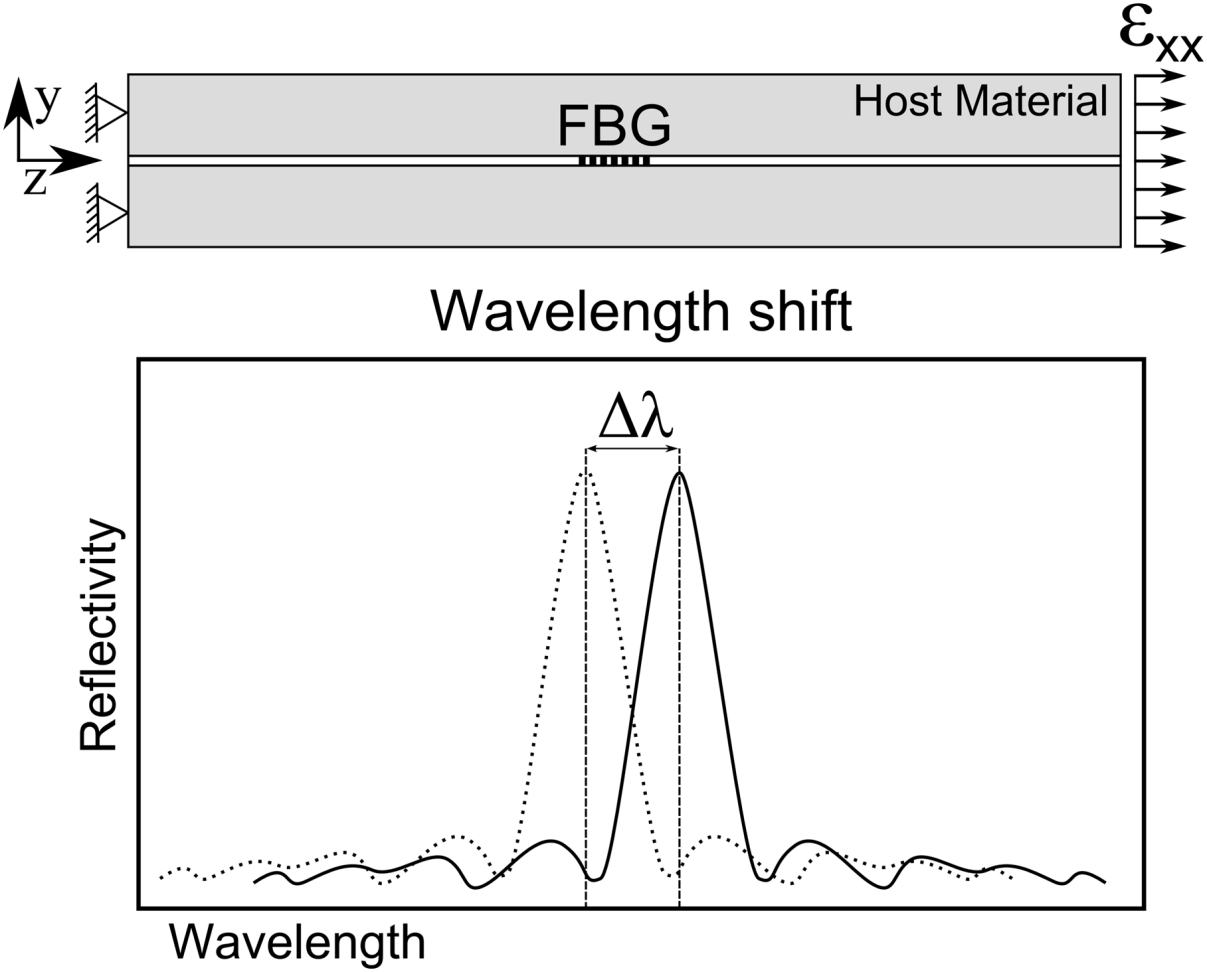
Embedded FBG response to a uniform variation of strain and/or temperature.

The sensor response to a uniform axial strain is schematically shown in Fig 5. Assuming a uniform strain *ɛ*_*xx*_ along the grating length, the wavelength shift Δ*λ*_*b*_ in the sensor response is described by Eq (3) [13].

$$\begin{array}{c} \frac{\Delta \lambda_{b}}{\lambda_{b}}=\left(1-p_{e}\right)\varepsilon_{xx} \end{array}$$ (3)

The parameter *p*_*e*_ is a photo-elastic coefficients.

#### Response to Transverse Deformation: Birefringence Effect

An optical fibre can exhibit birefringent behaviour, which is defined by the change in the refractive index *n*_*eff*_ of the two directions *n*_*effy*_ and *n*_*effz*_ when the grating is subjected to a transverse force [14–17]. The change in the refractive index of the two directions *n*_*effy*_ and *n*_*effz*_ is given by Eqs (4) and (5).

$$\begin{array}{c} \Delta n_{effz}=-\frac{n_{0}^{3}}{2E_{f}}\left\{\left(p_{11}-2\nu_{f}p_{12}\right)\sigma_{z}+\left[\left(1-\nu_{f}\right)p_{12}-\nu_{f}p_{11}\right]\left(\sigma_{y}+\sigma_{x}\right)\right\} \end{array}$$ (4)

$$\begin{array}{c} \Delta n_{effy}=-\frac{n_{0}^{3}}{2E_{f}}\left\{\left(p_{11}-2\nu_{f}p_{12}\right)\sigma_{y}+\left[\left(1-\nu_{f}\right)p_{12}-\nu_{f}p_{11}\right]\left(\sigma_{z}+\sigma_{x}\right)\right\} \end{array}$$ (5)

The parameter *E*_*f*_ is the elastic modulus of the optical fibre, *ν*_*f*_ is Poisson’s ratio, *n*_0_ is the initial refractive index, and *p*_11_ and *p*_12_ are the photo-elastic coefficients of the optical fibre.

**Fig 6:**
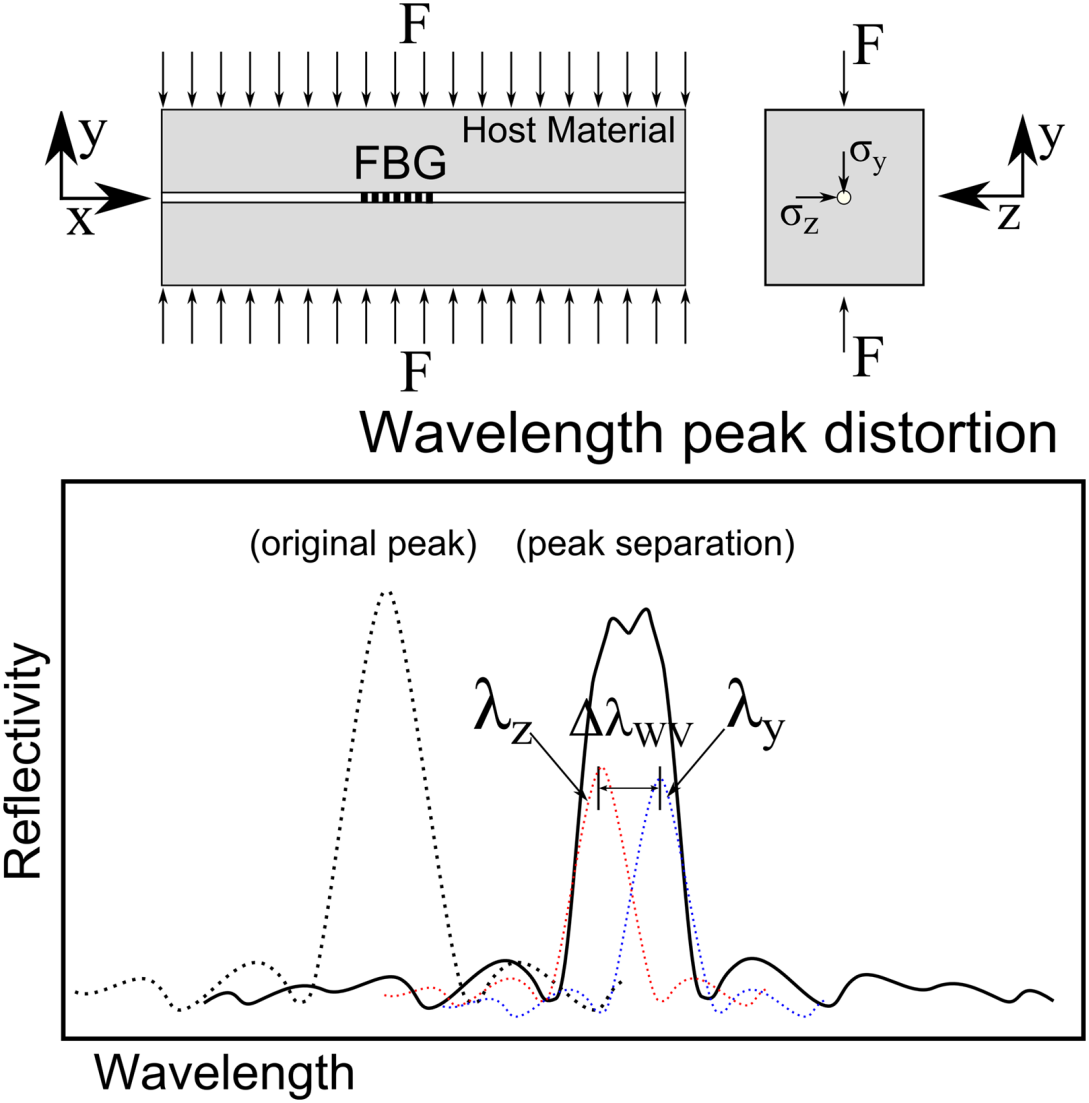
FBG response under a transverse force: Birefringent effect.

With this, when a transverse stress is applied to the grating, a separation of the reflected Bragg peak occurs (peak splitting), as presented in Fig 6. The width variation of the reflected peak due to transverse deformation [15] $\Delta \lambda_{WV}^{\prime }=|\lambda_{z}-\lambda_{y}|$ can be calculated using Eqs (1), (4) and (5).

$$\begin{array}{c} \Delta \lambda {{}^{\prime }}_{WV}=2\Lambda \left|\Delta n_{effz}-\Delta n_{effy}\right| \end{array}$$ (6)

$$\begin{array}{c} =\frac{\Lambda n_{o}^{3}}{E_{f}}\left[\left(1+\nu_{f}\right)\left(p_{12}-p_{11}\right)\right]\left|\sigma_{z}-\sigma_{y}\right| \end{array}$$ (7)

#### Response to Non-uniform Strain

**Fig 7:**
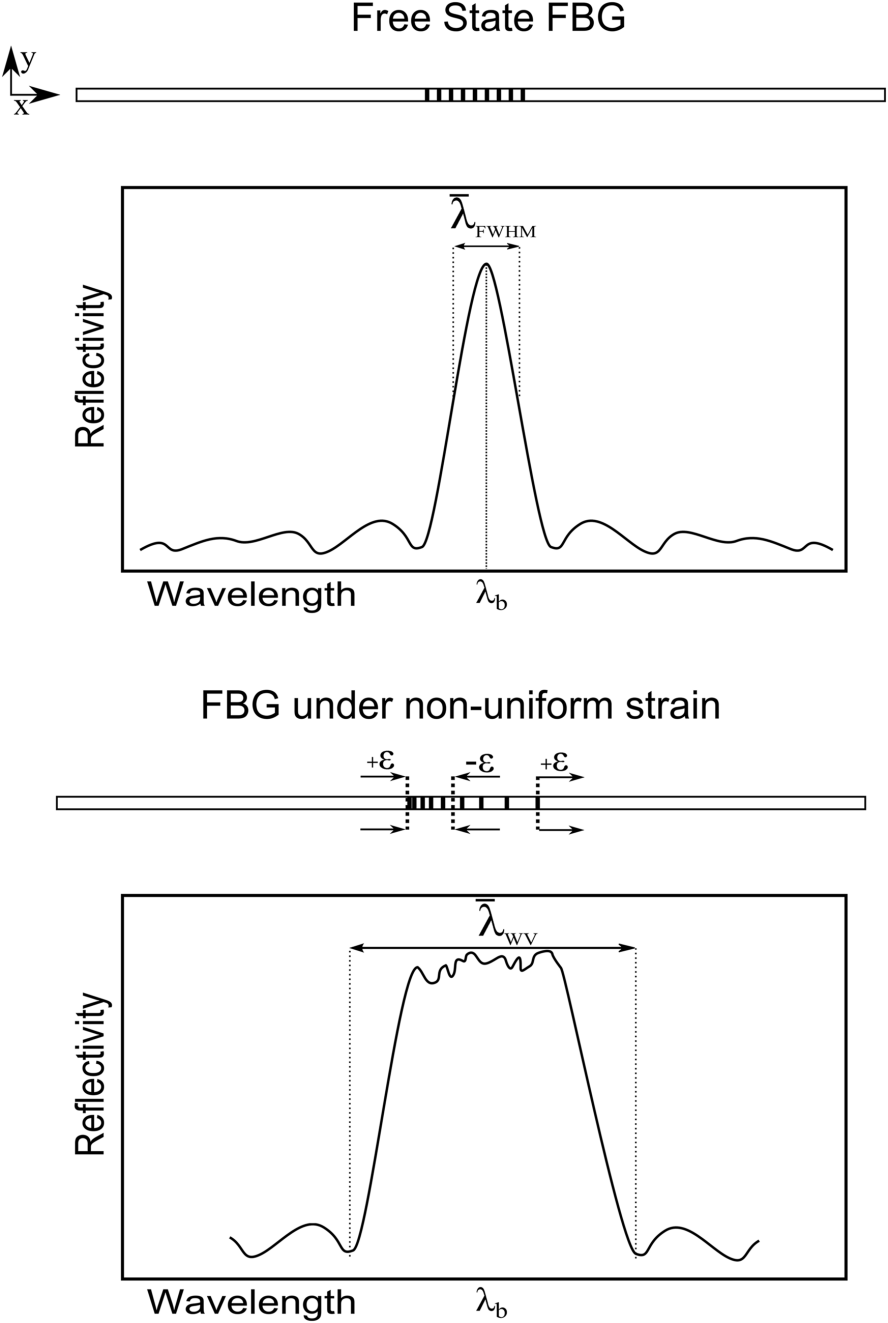
FBG response under a non-uniform strain.

A crack or defect in the material can create a stress concentration/gradient, which leads to an abrupt variation in strain. If the FBG sensor is inside this strain gradient zone, the grating will experience a non-uniform deformation, causing a sensor response that is significantly more complicated compared to a uniform case [14, 18]. The non-uniform strain along the sensor length will change the periodicity of the grating pattern. In this way, the grating pattern is modified from a *uniform* to a *chirped* configuration [19, 20], as shown in the Fig 7.

As demonstrated by Peters [12], in a non-uniform grating, the applied strain will induce a change in both the grating period and the mean index. These two effects can be superimposed by applying an effective strain of “(1 − *p*_*e*_)*ɛ*_*xx*_(*x*)”, where *ɛ*_*xx*_(*x*) is the strain variation along the *x* direction. Thus, it is possible to rewrite the grating period from Eq (1) as [12].

$$\begin{array}{c} \Lambda \left(x\right)=\Lambda_{0}\left[1+\left(1-p_{e}\right)\varepsilon_{xx}\left(x\right)\right] \end{array}$$ (8)

The effective mode index along the *x* direction *δn*_*eff*_(*x*) can be calculated by [12].

$$\begin{array}{c} \delta n_{eff}\left(x\right)=(1+\xi cos[\frac{2\pi }{\Lambda_{o}\left[1+\left(1-p_{e}\right)\varepsilon_{xx}\left(x\right)\right]}z]) \end{array}$$ (9)

In a real crack growth situation, the strain variation along the grating *ɛ*_*xx*_(*x*) (non-uniform strain) can be difficult to predict/simulate due to its strong non-linearity. The authors propose a simple method for evaluating the contribution of the non-uniform strain to the width variation of the reflected peak by subtracting the bandwidth of the grating in a free state $\bar{\lambda_{0}}$ from the bandwidth $\bar{\lambda_{WV}}$ of the grating under a linear variation of strain calculated using the maximum ${ɛ}_{xx}^{max}(x)$ and minimum ${ɛ}_{xx}^{min}(x)$ strains along the grating length.

The bandwidth of an FBG in a free state can be calculated using an approximate expression that provides the full-width at half-maximum (FWHM) bandwidth [21],

$$\begin{array}{c} \bar{\lambda_{0}}\approx \bar{\lambda_{FWHM}}=\lambda_{b}s{\left[{\left(\frac{\delta n_{eff}}{2n_{core}}\right)}^{2}+{\left(\frac{\Lambda }{L}\right)}^{2}\right]}^{1/2} \end{array}$$ (10)

where *s* ≈ 1 for strong gratings with high reflectivity, and *s* ≈ 0.5 for weak gratings, *n*_*core*_ is the unexposed core refractive index.

The width variation of the reflected peak resulting from the non-uniform strain effect can be approximated using the maximum and minimum strain values along the grating, ${ɛ}_{xx}^{max}(x)$ and ${ɛ}_{xx}^{min}(x)$, respectively. The maximum grating period Λ_*max*_ and minimum grating period Λ_*min*_ can be calculated using Eq (8), and the width variation of the reflected peak resulting from non-uniform strain $\Delta \lambda_{WV}^{\prime \prime }$ is obtained by combining Eqs (8) and (1).

$$\begin{array}{c} \Delta \lambda {{}^{\prime \prime }}_{WV}=\left[2n_{eff}\Lambda_{max}-2n_{eff}\Lambda_{min}\right]-\bar{\lambda_{FWHM}} \end{array}$$ (11)

### FBG Response During Crack Growth

**Fig 8:**
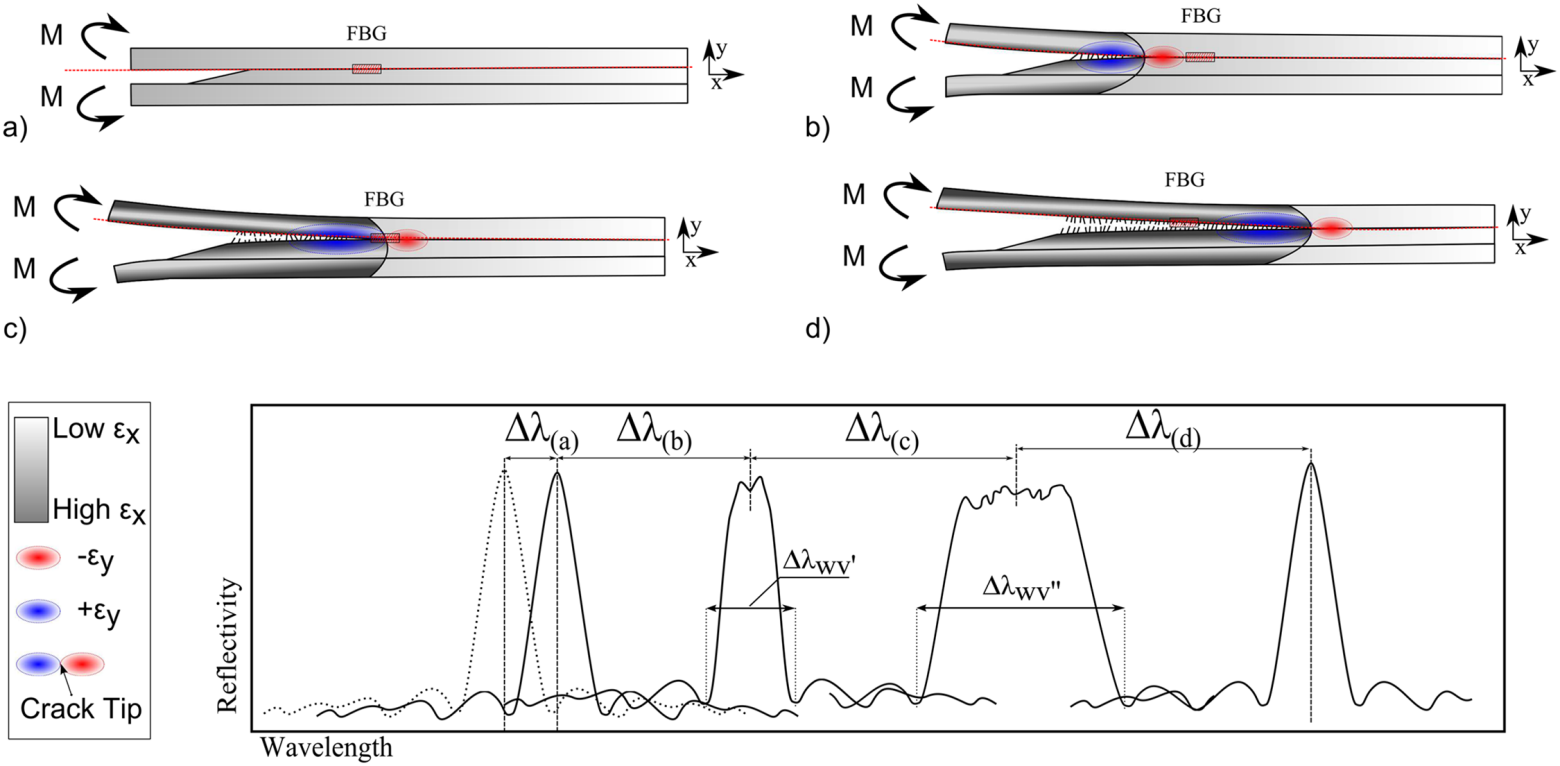
Different stages of the FBG response under a crack growth event.

The FBG responses under different stages of crack growth are shown in Fig 8.

8(a) - No crack is present and a uniform strain, *ɛ*_*xx*_, builds up around the grating area as the structure is loaded. The FBG response is a uniform wavelength shift in the reflected peak, Δ*λ*.

8(b) - A crack has initiated and is approaching the grating area. A compressive strain transverse to the FBG, *ɛ*_*yy*_, forms ahead of the crack tip. Compressive strain changes the FBG response creating a splitting (and hence a widening) of the reflected peak, $\Delta \lambda_{WV}^{\prime }$.

8(c) - Progression of the crack causes a non-uniform strain field around the crack tip to reach the grating area. This modifies the FBG response by significantly increasing the width of the reflected peak, $\Delta \lambda_{WV}^{\prime \prime }$.

8(d) - The crack has passed the FBG sensor, and the FBG response has returned to its original shape. Only uniform strain acts on the grating, resulting in a uniform shift of the FBG reflected peak, Δ*λ*.

## Finite Element Method Model

### Delamination Model

To analyse the delamination problem and to link it with the structural health monitoring technique, a finite element method (FEM) model of a double cantilever beam (DCB) specimen was developed using the commercial software ABAQUS. This specimen geometry, DCB, was chosen because it is commonly used in fracture testing of composite materials, and later in this article, it will be used for experimental validation. The model was developed assuming plane stress conditions (plane stress elements), and the delamination/fibre bridging was modelled using 4-node cohesive elements along the delamination plane [22, 23].

**Fig 9:**
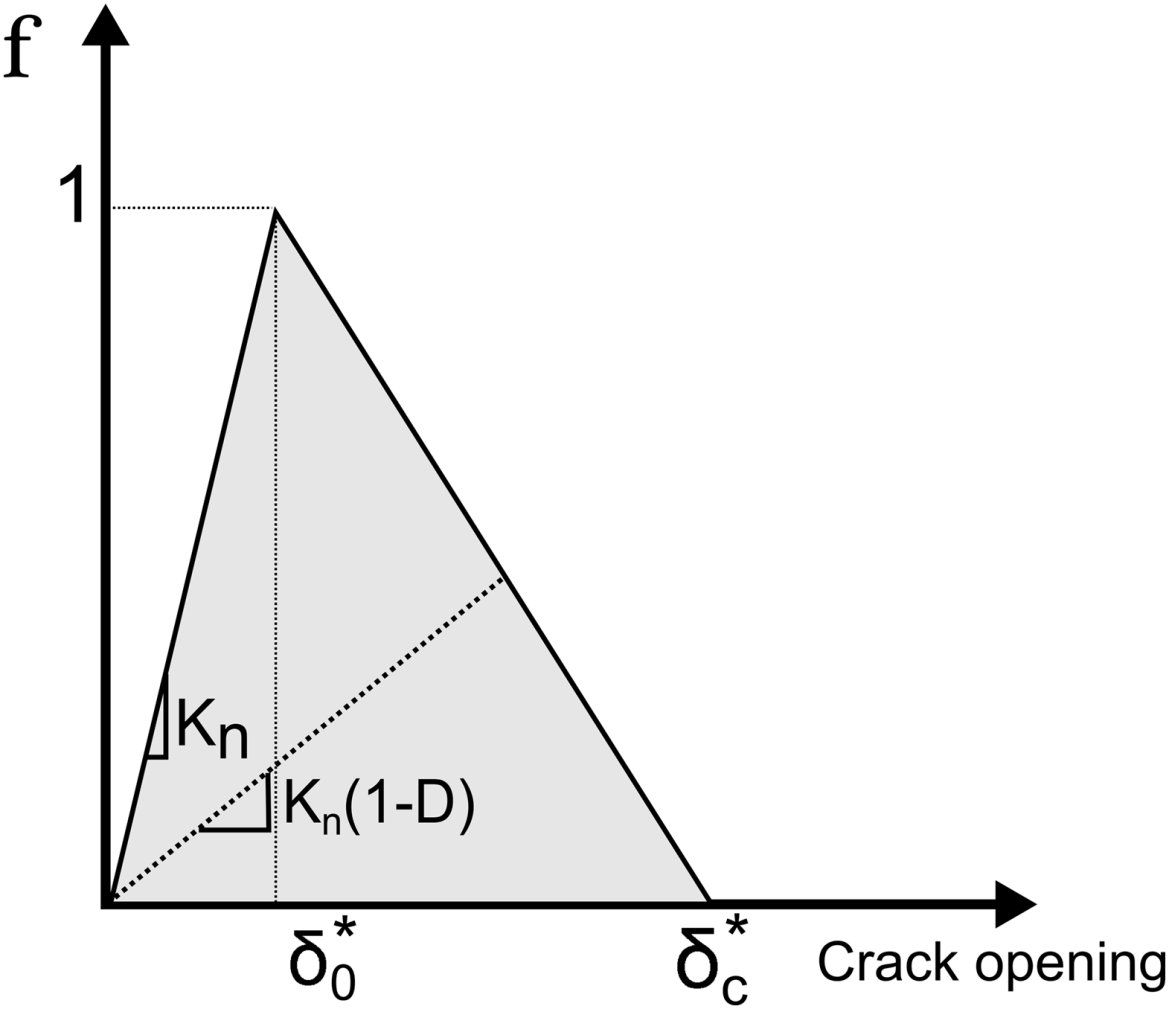
Constitutive behaviour of the cohesive element.

This method assumes that one or more interface elements (cohesive elements) can be predefined to hold the delamination phenomenon, allowing the introduction of a discontinuity in the displacement field. The cohesive elements are modelled to express the cohesive law (traction-separation), meaning a progressive loss of the cohesion between the two crack faces with the local crack opening *δ*. The crack was modelled to occur between the interface of the adhesive and the glass fibre arm beam. A cohesive element with a small thickness (0.5% of the adhesive layer thickness) was used to model only the interface between the two materials and to avoid neglecting the elastic contribution of the adhesive to the DCB global behaviour. In an undamaged state, the cohesive element follows a linear-elastic behaviour, defined as the penalty stiffness *K*_*n*_, which relates the nominal stress (traction vector- *σ*_*n*_, *σ*_*s*_, *σ*_*t*_) to the nominal strains (*δ*_*n*_, *δ*_*s*_, *δ*_*t*_), as presented in Fig 9.

The damage initiation was calculated using a quadratic stress criterion presented in Eq (12).[24]

$$\begin{array}{c} f={\left(\frac{\sigma_{n}}{N_{max}}\right)}^{2}+{\left(\frac{\sigma_{s}}{S_{max}}\right)}^{2}+{\left(\frac{\sigma_{t}}{T_{max}}\right)}^{2}=1 \end{array}$$ (12)

The parameter *f* is the damage criterion, and it is fulfilled when it reaches the value *f* = 1. The parameters *σ*_*n*, *s*, *t*_ are the nominal stress in the normal, first shear and second shear directions, respectively, and *N*_*max*_, *S*_*max*_, and *T*_*max*_ are cohesive law parameters; these parameters are determined experimentally. The parameters $\delta_{0}^{*}$ and $\delta_{c}^{*}$ are the crack opening displacement to the local crack plane for damage initiation and critical damage. For mixed mode loading, $\delta_{0}^{*}$ and $\delta_{c}^{*}$ were calculated using the law of Pythagoras.

$$\begin{array}{c} \delta_{0}^{*}=\sqrt{\delta_{0,n}^{2}+\delta_{0,s}^{2}}\;;\;\delta_{c}^{*}=\sqrt{\delta_{c,n}^{2}+\delta_{c,s}^{2}} \end{array}$$ (13)

When the initiation criterion is reached, a damage evolution law will describe the material stiffness degradation. A scalar damage variable, D, ranging from 0 (no damage) to 1 (fully damaged), represents the damage in the cohesive element. A linear softening displacement criterion was used, given by *δ*_0_, which is the opening at damage initiation, and *δ*_*c*_, which is the opening at failure. In terms of mixed mode behaviour, a linear relation between Modes I and II was implemented.

#### Description of the Delamination FEM Model

**Fig 10:**
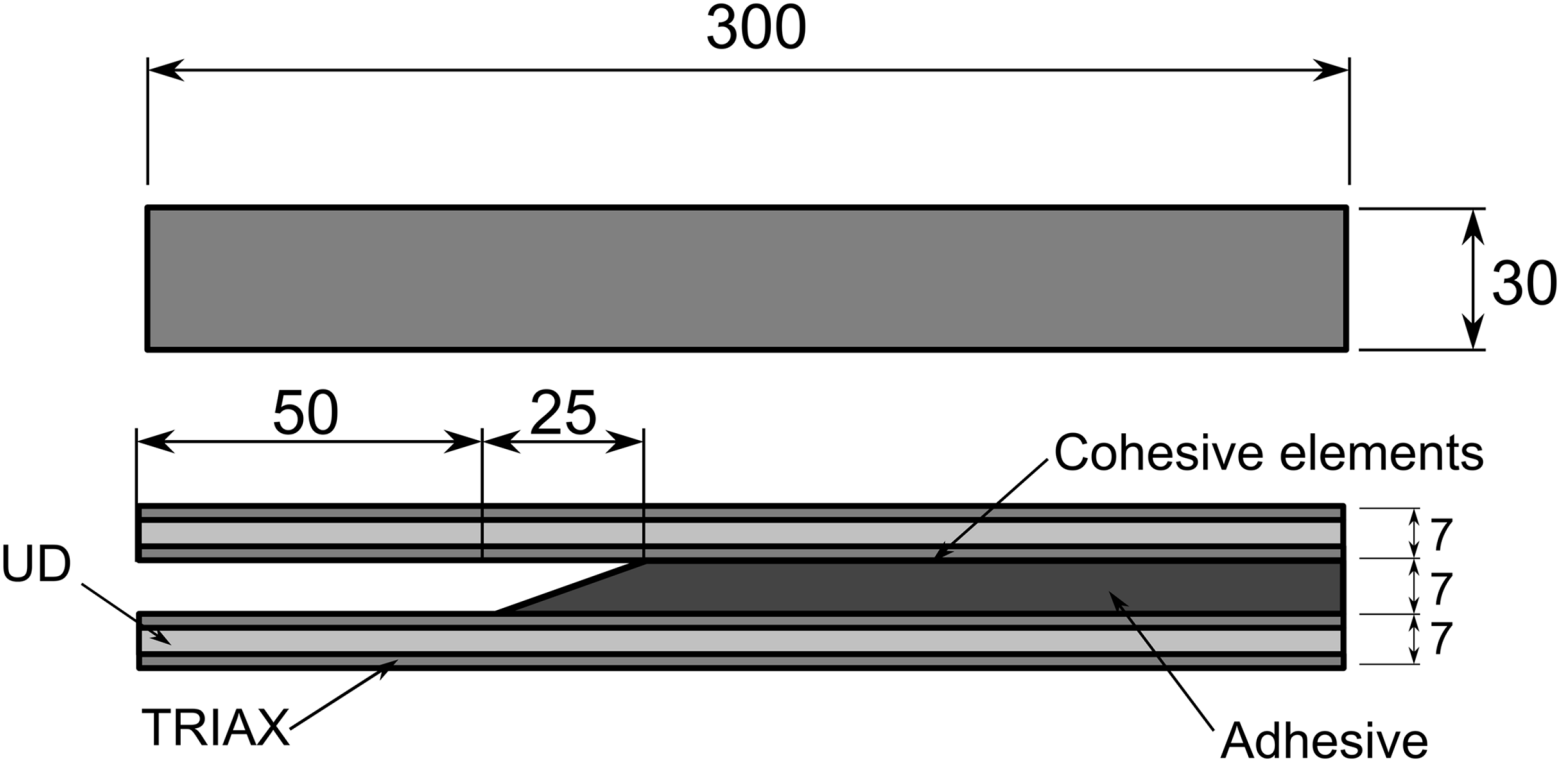
Double cantilever beam geometry dimensions.

The dimensions of the DCB specimen are shown in Fig 10, and the material properties implemented in the FEM model are presented in Table 1.

**Table 1:** Double cantilever beam material properties.

**Composite Material**		**Adhesive**
Triaxial Fabric (Composite)	Uniaxial Fabric (Composite)	Elastic
*E*_1_ = 44.3 GPa	*E*_1_ = 23.8 GPa	*E* = 4.56 GPa
*E*_2_ = *E*_3_ = 12.9 GPa	*E*_2_ = *E*_3_ = 15.05 GPa	*ν* = 0.35
*ν*_12_ = *ν*_13_ = *ν*_23_ = 0.23	*ν*_12_ = *ν*_13_ = *ν*_23_ = 0.513	
*G*_12_ = *G*_13_ = *G*_23_ = 4393 GPa	*G*_12_ = *G*_13_ = *G*_23_ = 4.393 GPa	
**Interface (Cohesive Law)**		
Penalty Stiffness	Damage (Quadratic stress)	Damage Evolution
*K* = 4.2 *E*12 Pa;	*σ*_*n*_ = 2.64 MPa (Mode I)	*δ*_*c*1_ = 1.4 (Mode I)
	*σ*_*t*_ = 22.15 MPa (Mode II)	*δ*_*c*2_ = 0.37 (Mode II)

**Fig 11:**
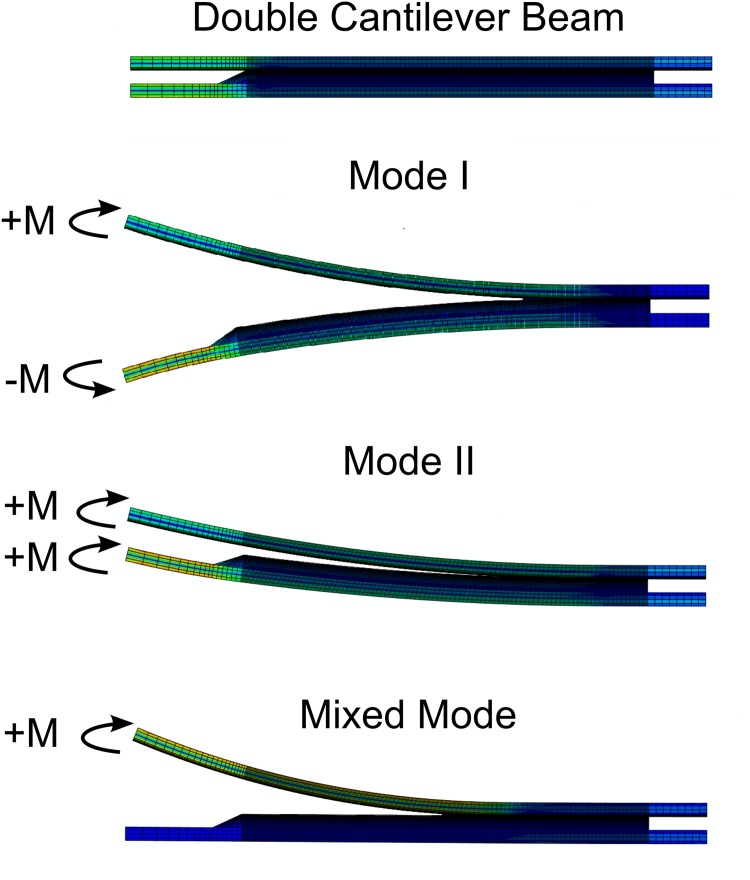
FEM simulation of different fracture modes in a DCB specimen.

The beams were modelled using a combination of two different laminates: unidirectional glass fibre (UD) and triaxial glass fibre (Triax). Moments were applied to the extremities of the beams, as shown in Fig 11. Three different loading combinations were used: pure mode I-opening fracture, by applying identical moments to the DCB arms; pure mode II-shear fracture, by applying symmetric moments to the DCB arms; and mixed mode-opening and shear fracture, by applying a moment to one arm and leaving the other arm free.

**Fig 12:**
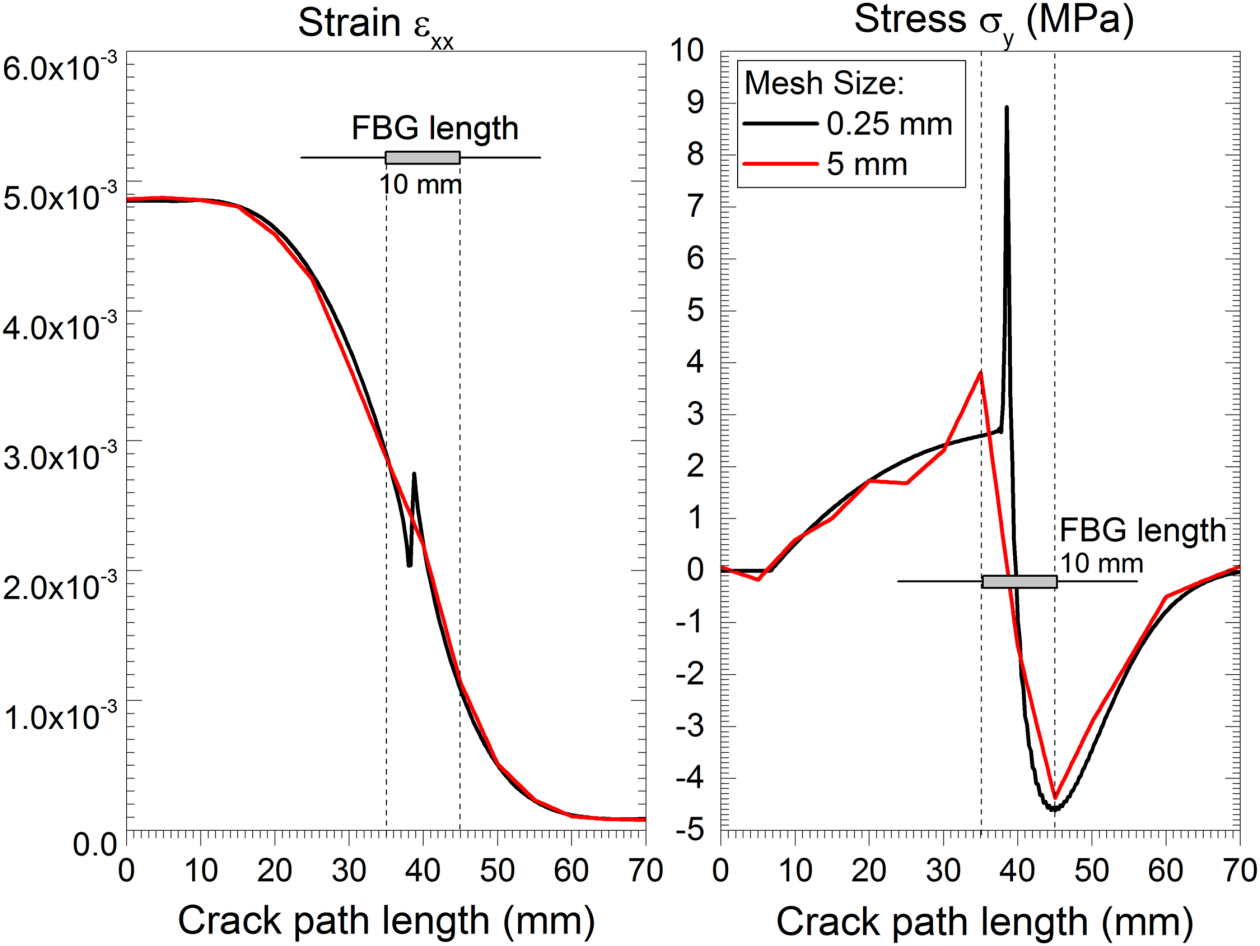
Mesh resolution study: cohesive zone and the stress/strain variation along the grating length.

A minimum of 10 cohesive elements inside the active fracture process zone is suggested by some authors [25, 26]. Using too few elements will introduce error in the crack growth resistance (fracture energy) calculation; however, finer meshes require more computational resources. Moreover, the mesh should be sufficiently fine to accurately represent the cohesive zone and the stress/strain variation along the grating length. An example of a coarse mesh is shown in Fig 12. The stress and strain are not correctly represented along the grating length, leading to an inaccurate prediction of the sensor output.

A mesh resolution and result convergence study was conducted, as presented in Table 2. The maximum stress *σ*_*y*_ that is possible to measure along the sensor length (*10 mm*) and the maximum strain variation Δ*ɛ*_*xx*_ at the crack tip were analysed. Based on this analysis, a cohesive element size of 0.5 *mm* was selected, which meets the minimum element number requirement and provides a good stress and strain resolution along the sensor length.

**Table 2:** Mesh resolution and result convergence study.

	Mode I		Mode II	
Element size (mm)	Max *σ*_*y*_ (MPa)	Max Δ*ɛ*_*xx*_ (%)	Max *σ*_*y*_ (MPa)	Max Δ*ɛ*_*xx*_ (%)
**5.0**	3.81	0	1.29	0
**3.8**	3.78	0	2.36	0.012
**1.0**	6.27	0.2	5.55	0.022
**0.5**	7.84	0.7	10.13	0.82
**0.25**	7.88	0.71	10.67	0.87

### FBG Response Model: Crack Detection/Prediction

One of the goals of this article is to develop a numerical model for predicting the FBG output in a general crack growth situation, thus making it possible to use this *material-structure-sensor* model as a design tool, and to study the application of this monitoring technology in different composite material structures/locations.

**Fig 13:**
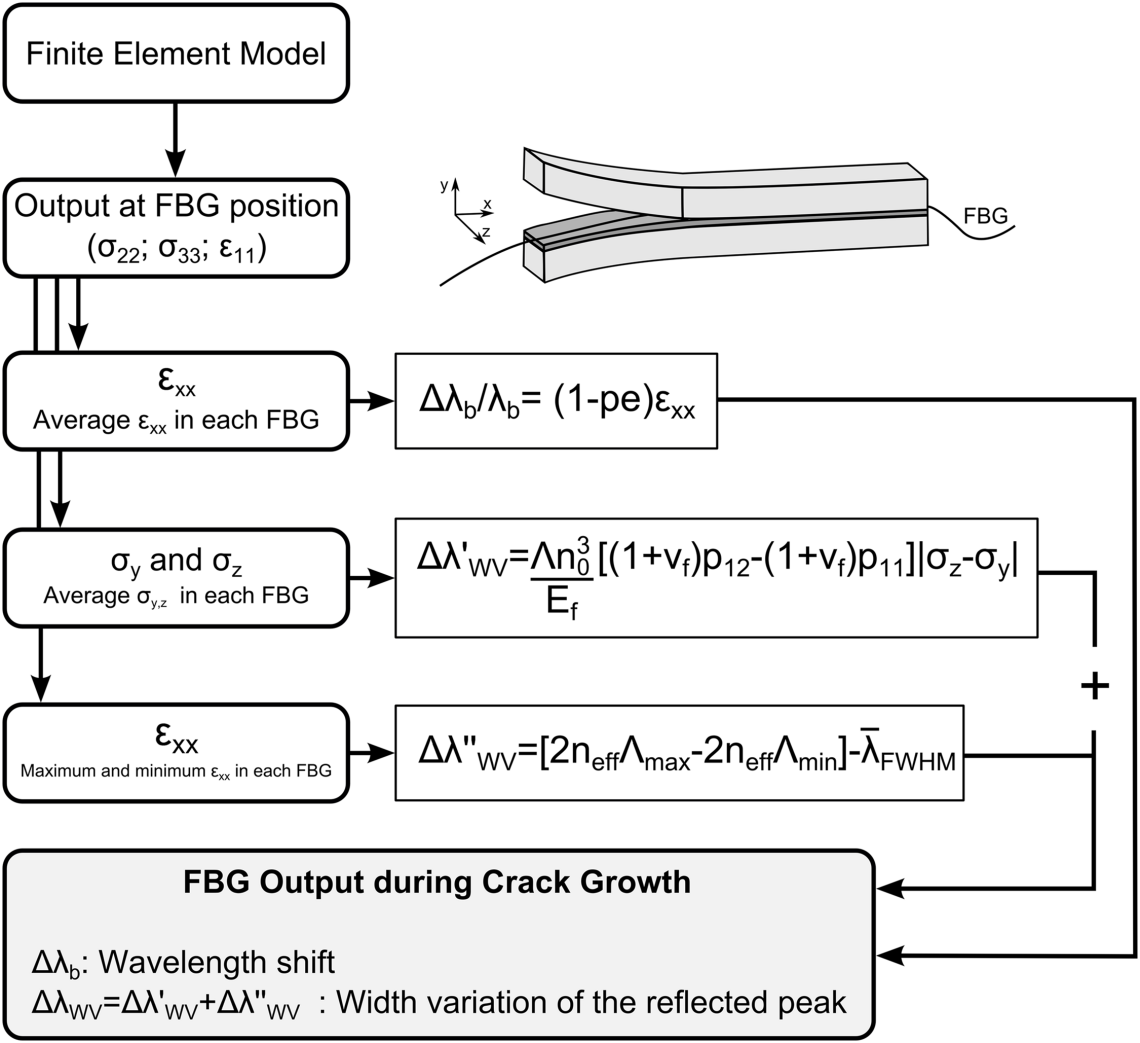
Algorithm applied to the FEM model to obtain the FBG output prediction.

To accomplish this goal, an algorithm was developed using *Python* and incorporated into the FEM model, as shown in Fig 13. The algorithm was developed as a post-processing tool that uses the stress *σ* and strain *ɛ* state at the grating positions as input. In the first step, the algorithm synchronises the stress, the strain, and the crack tip position with the virtual grating positions. Then, the algorithm computes the wavelength shift Δ*λ* and width variation of the reflected peak Δ*λ*_*WV*_ versus the crack position for each virtual grating using the equations developed in this article.

Some assumptions were made to compute the contribution of each fracture phenomenon to the sensor response. In a real application, the grating has a finite length, generally 8–10 mm; however, the FEM technique discretised the grating into finite parts or elements. Thus, to compute the wavelength shift Δ*λ*_*b*_, which only depends on the global state of strain *ɛ*_*xx*_ around the grating, the average strain *ɛ*_*x*_ in the elements at the position of the virtual grating was used. Similarly, to compute the width variation of the reflected peak due to the compressive strain $\Delta \lambda_{WV}^{\prime }$, the average stress in the transverse direction *σ*_*y*_ and *σ*_*z*_ at the virtual grating position was used. To compute the non-uniform strain contribution to the reflected peak width variation $\Delta \lambda_{WV}^{\prime \prime }$, a linear strain variation was assumed, using the maximum and minimum strain values along the elements in the virtual grating position. In reality, the strain distribution follows a polynomial curve, which depends on the material, geometry and crack shape. However, this approximation can be considered good due to the small size of the sensor. The final width variation Δ*λ*_*WV*_ value is obtained by simply adding both contributions: the non-uniform strain ($\Delta \lambda_{WV}^{\prime \prime }$) and the transverse stress ($\Delta \lambda_{WV}^{\prime }$). The *Python* script used to calculate the sensor response from the FEM model is shown in Supporting information (S1 File).

#### Description of the Sensor Response FEM Model

**Fig 14:**
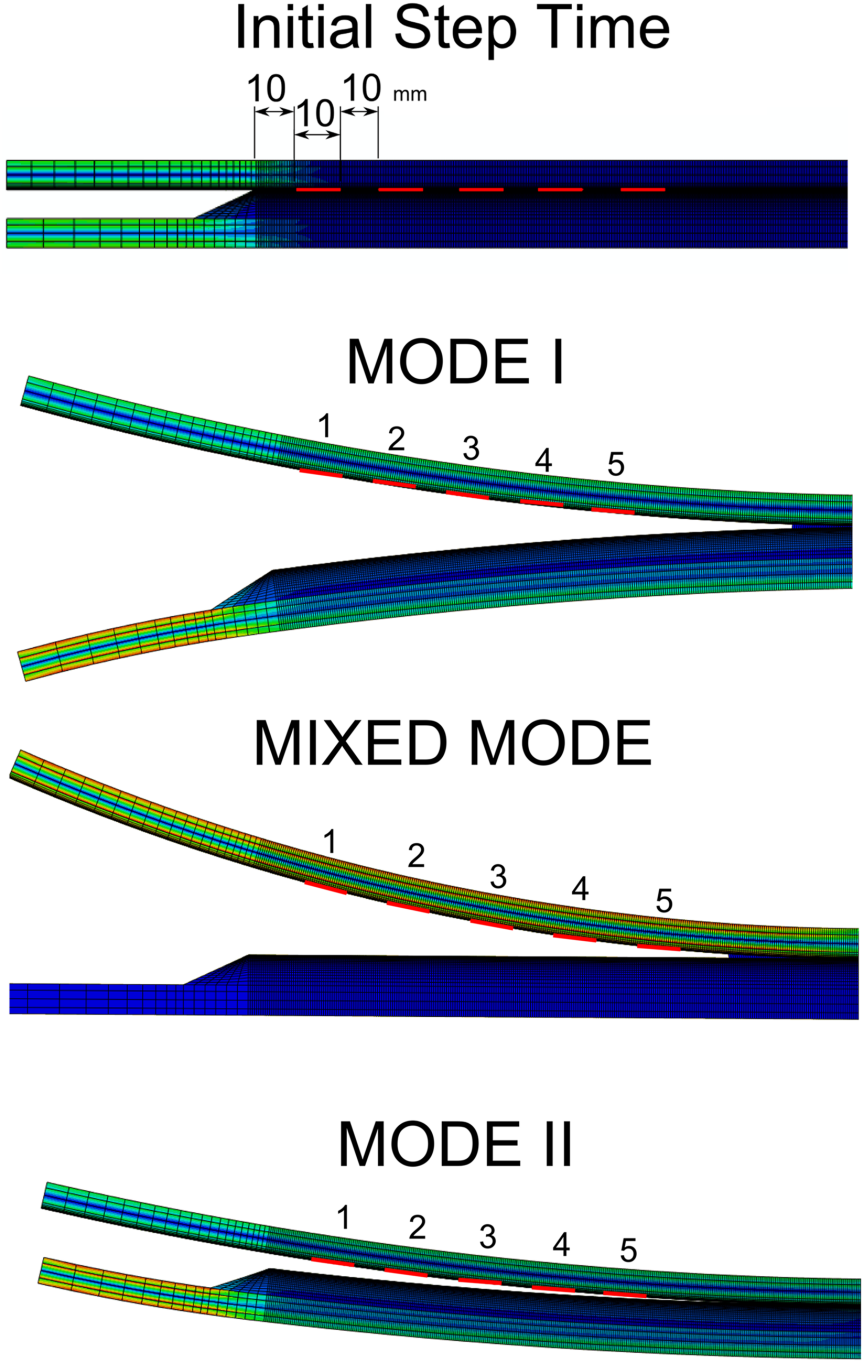
FBG measurement point in the FEM model.

In the FEM model, pure mode I, pure mode II and mixed mode were simulated to represent different crack growth conditions. An FBG array of 5 gratings was defined as virtual measurement points, each with a *10 mm* length and spaced *10 mm* from each other. The first grating was placed 10 mm from the beginning of the adhesive, as shown in Fig 14. The FBG array was placed between the interface of the composite material and the structural adhesive.

Table 3 lists the parameters of the optical fibre used to implement the algorithm.

**Table 3:** Fibre Bragg Grating Parameters.

Parameters:	
*λ*_*b*_- Initial wavelength	From manufacturer (Ex:1528.81;1541.31; 1554.25;1567.12; 1580.24 (nm))
L- FBG length	10 (mm)
*n*_*eff*,0_- Initial refractive Index	1.45
*p*_*e*_- Photo-elastic coefficient	0.215
*p*_11_- Photo-elastic coefficient [20]	0.121
*p*_12_- Photo-elastic coefficient [20]	0.270
*E*_*f*_- Elastic modulus of FBG	75 GPa
*ν*_*f*_- Poisson’s ratio of FBG	0.17

## Numerical Simulation of the FBG Sensor Output During Crack Growth

**Fig 15:**
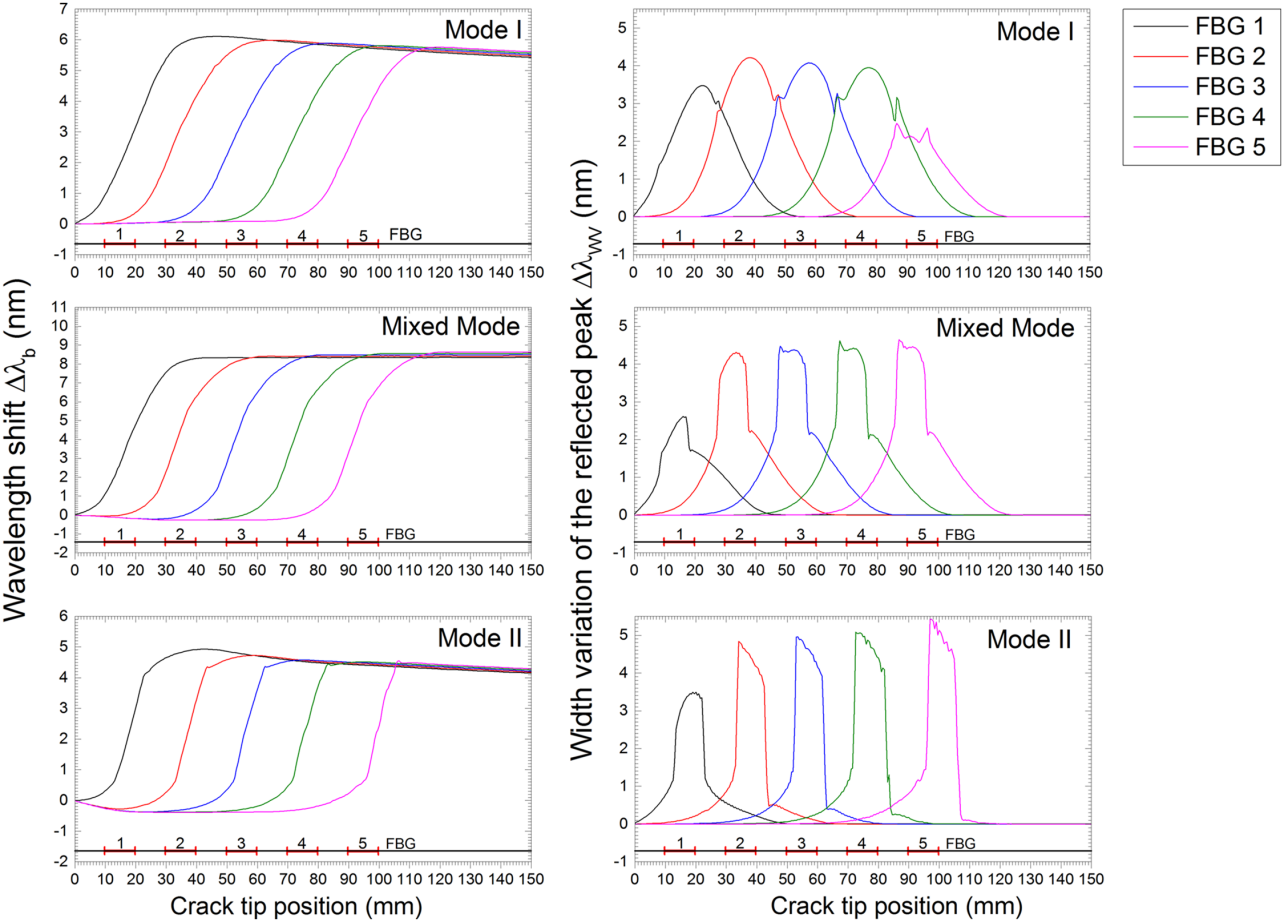
FBG sensor output simulation under crack growth: Mode I, II and mixed Mode fracture.

In Fig 15, the numerical simulation of the FBG output response for a crack growing in a DCB specimen is shown.

The plots in the left column represent the wavelength shift Δ*λ*_*b*_ caused by the longitudinal strain *ɛ*_*xx*_. The plots in the right column represent the width variation of the reflected peak Δ*λ*_*WV*_ caused by the fracture/damage phenomenon near the grating. Different fracture modes were addressed, namely, Mode I, Mixed Mode and Mode II in the first, second and third plot rows, respectively. The wavelength shift Δ*λ*_*b*_ and the width variation of the reflected peak Δ*λ*_*WV*_ vs. crack tip position (CTP) were plotted in all figures. At each abscissa point (CTP), the output values of the 5 FBG sensors in that specific crack position are presented.

A jump in the wavelength shift Δ*λ*_*b*_ was observed when the crack passed the position of the grating. The damage/crack changes the local compliance of the material and load distribution, making the area that surrounds the sensor less stiff and more deformed; therefore, an increase in the strain was measured. However, it is possible to observe some differences in the evolution (shape) of the wavelength shift Δ*λ* because the position of the sensor and the crack related to the applied moments is different.

The model predicted that a variation in the width of the reflected peak Δ*λ*_*WV*_ will occur when the crack is near the grating, in which the original peak width is restored after the crack passes the grating. The width variation response in all cases showed the same evolution pattern, exhibiting a loading- and geometry-independent behaviour. The magnitude of the differences of the Δ*λ*_*WV*_ in mode II is related to the fracture material properties, i.e., the fracture resistance in mode II is higher than that in mode I. This means that the stress distribution during crack growth in mode II is different, creating a higher strain variation. The differences observed in the Δ*λ*_*WV*_ response for the FBG *5* in Mode I, FBG *1* in Mode II, and FBG *1* in Mixed Mode are due to the effect of the model boundary conditions.

**Fig 16:**
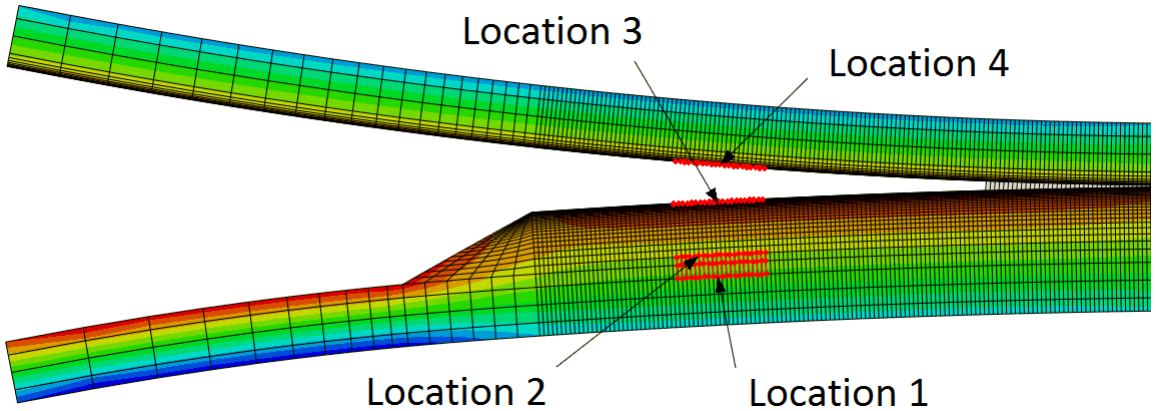
FBG sensor position analysis scheme.

**Fig 17:**
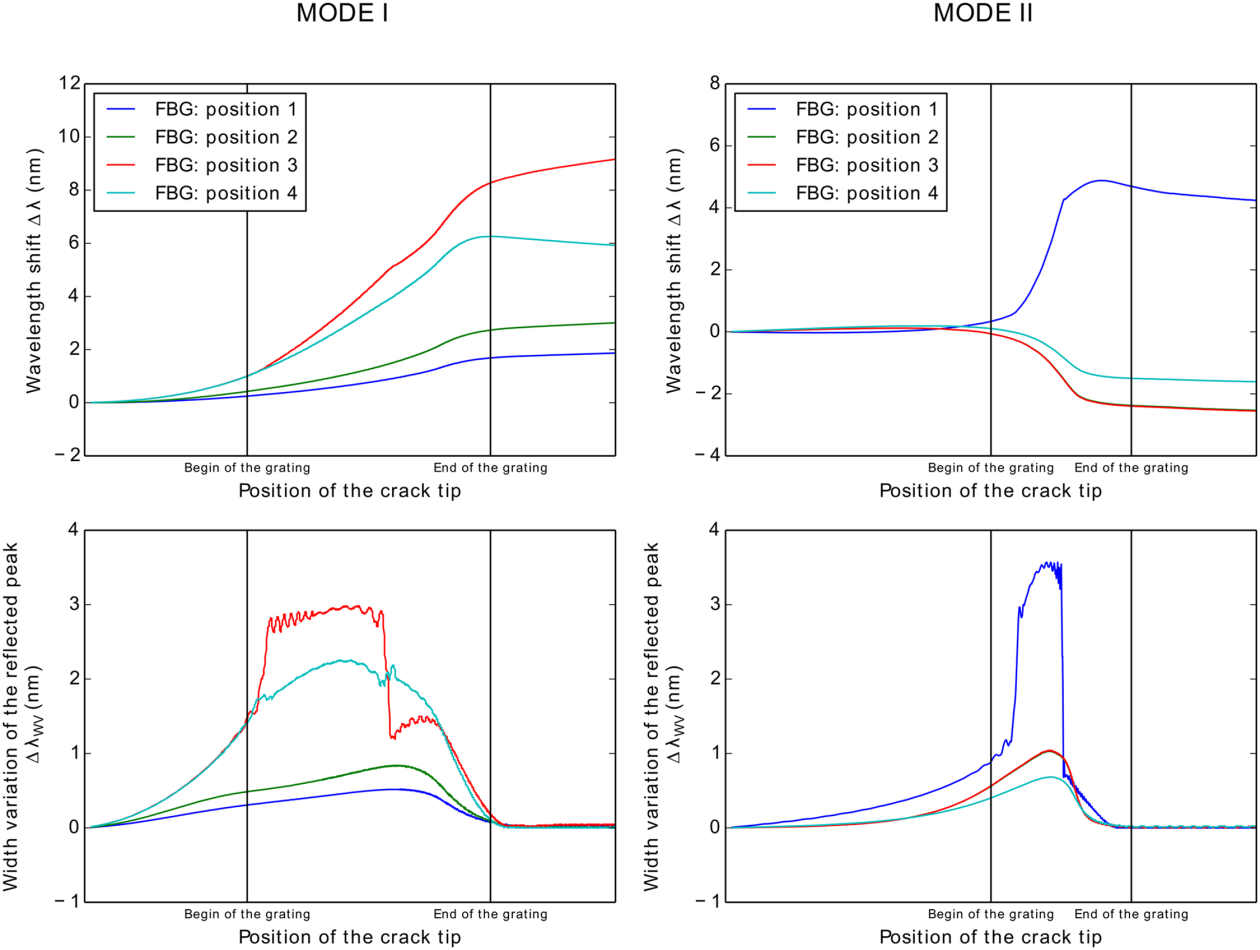
FBG sensor position analysis. a) Sensor output for Mode I fracture. b) Sensor output for Mode II fracture.

As previously discussed, this *material-structure-sensor* can be used as a tool for studying the application of this monitoring technology in different locations or structures. A grating position analysis scheme is presented in Fig 16. Four positions were analysed: bottom composite laminate, bottom adhesive-composite interface, top adhesive-composite interface, and top composite laminate for Mode I and Mode II fractures. The sensor response for each position is shown in Fig 17. As expected, in Mode I fracture (Fig 17a), the gratings located closer to the crack tip measure higher magnitude values. However, placing the sensor close to the crack can be technically difficult or even increase the probability of damaging the sensor during crack growth.

This analysis revealed that the sensor in position 2 (interface) can confidently detect damage because the common resolution of measurement equipment is approximately 0.01 *nm* and ensure the structural integrity of the sensor by increasing the distance from the crack surface.

In Mode II fracture (Fig 17b), the sensor in position 1 showed a greater magnitude of Δ*λ*_*b*_ and Δ*λ*_*WV*_. This result is because at position 1, the sensor is more distant from the bending neutral-axis, consequently deforming more, *ɛ*_*xx*_ → Δ*λ*_*b*_, and experiencing a larger amount of non-uniform strain, *ɛ*_*xx*_(*x*) → Δ*λ*_*WV*_.

Note that the sensor output Δ*λ*_*WV*_ showed a variation in the signal that depends on the location of the crack and the loading type. However, for the width variation of the reflected peak Δ*λ*_*WV*_, the sensor showed the same behaviour for different fracture modes, presenting a loading-independent behaviour. This makes **Δ** *λ*_**WV**_ **a key parameter for detecting cracks** in composite material structures.

## Model Experimental Validation

### Material and Experimental Procedure

To measure the FBG sensor response under a crack/delamination situation, double cantilever beam specimens with embedded fibre Bragg grating sensors were subjected to a controlled fracture progression. A special effort was made to identify specific fracture features, such as compression stress and non-uniform fields, during the crack growth.

#### Loading and Fracture Modes

**Fig 18:**
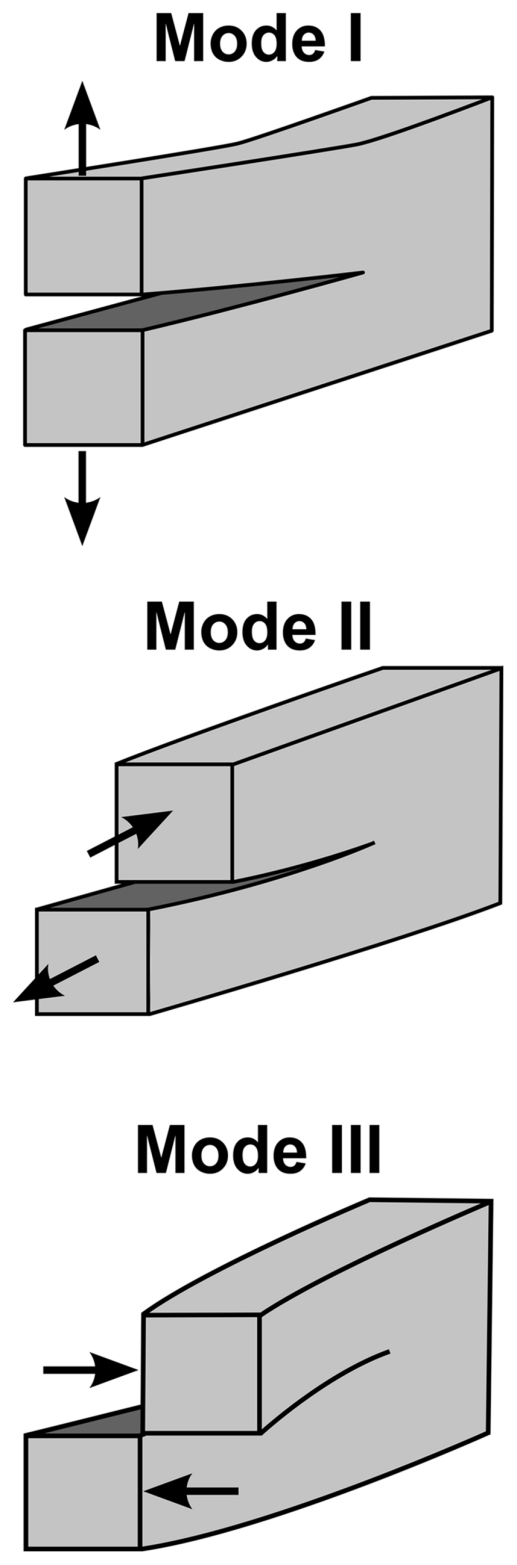
Scheme of the three modes of loading that can be applied to a crack.

The three different combinations of forces that can cause a crack to grow are presented in Fig 18. Mode I crack: opening mode, by tensile stress normal to the plane of the crack. Mode II crack: shear mode, by shear stress acting parallel to the plane of the crack and perpendicular to the crack front. Mode III: tearing mode, by shear stress acting parallel to the plane of the crack and parallel to the crack front.

Nominal mode mixity or phase angle, *ψ*_*nom*_, is a parameter that defines the ratio of Mode I and Mode II [27],

$$\begin{array}{c} \psi_{nom}=tan^{-1}(\frac{K_{II}}{K_{I}}) \end{array}$$ (14)

where *K*_*II*_ and *K*_*I*_ are the mode II and mode I stress intensity factors, respectively.

**Fig 19:**
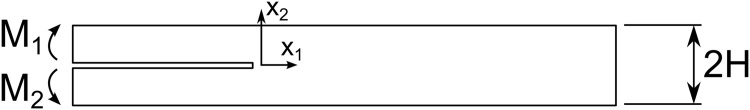
Homogeneous mixed mode specimen scheme.

For a homogeneous specimen without considering the adhesive layer, as shown in Fig 19, the parameter *ψ*_*nom*_ can be defined as [28]

$$\begin{array}{c} \psi_{nom}=tan^{-1}(\frac{\sqrt{3}}{2}\frac{M_{1}+M_{2}}{M_{1}+M_{2}}) \end{array}$$ (15)

where *M*_1_ and *M*_2_ are the moments applied to the top and bottom arms, respectively. The mixed mode ratio *ψ*_*nom*_ can be defined by the ratio of the moments applied to the DCB arms (*M*_1_/*M*_2_). For pure Mode I (opening fracture *ψ*_*nom*_ = 0°), when the moments are the same and applied in opposite directions, *M*_1_ > *M*_2_ and ∥ *M*_1_ ∥ = ∥ *M*_2_ ∥, and for pure Mode II (shearing fracture *ψ*_*nom*_ = 90°), when the moments are the same and applied in the same direction, *M*_1_ = *M*_2_.

The three different fracture modes used to conduct the experiments were as follows: pure Mode I, opening fracture *ψ*_*nom*_ = 0°; pure Mode II, shear fracture *ψ*_*nom*_ = 90°; and mixed mode I/II, with a phase angle of *ψ*_*nom*_ = 68°.

The experiments were conducted with a constant displacement rate of the lower beam of the test machine of 2.5 mm/min [29].

#### Fracture Testing Procedure

**Fig 20:**
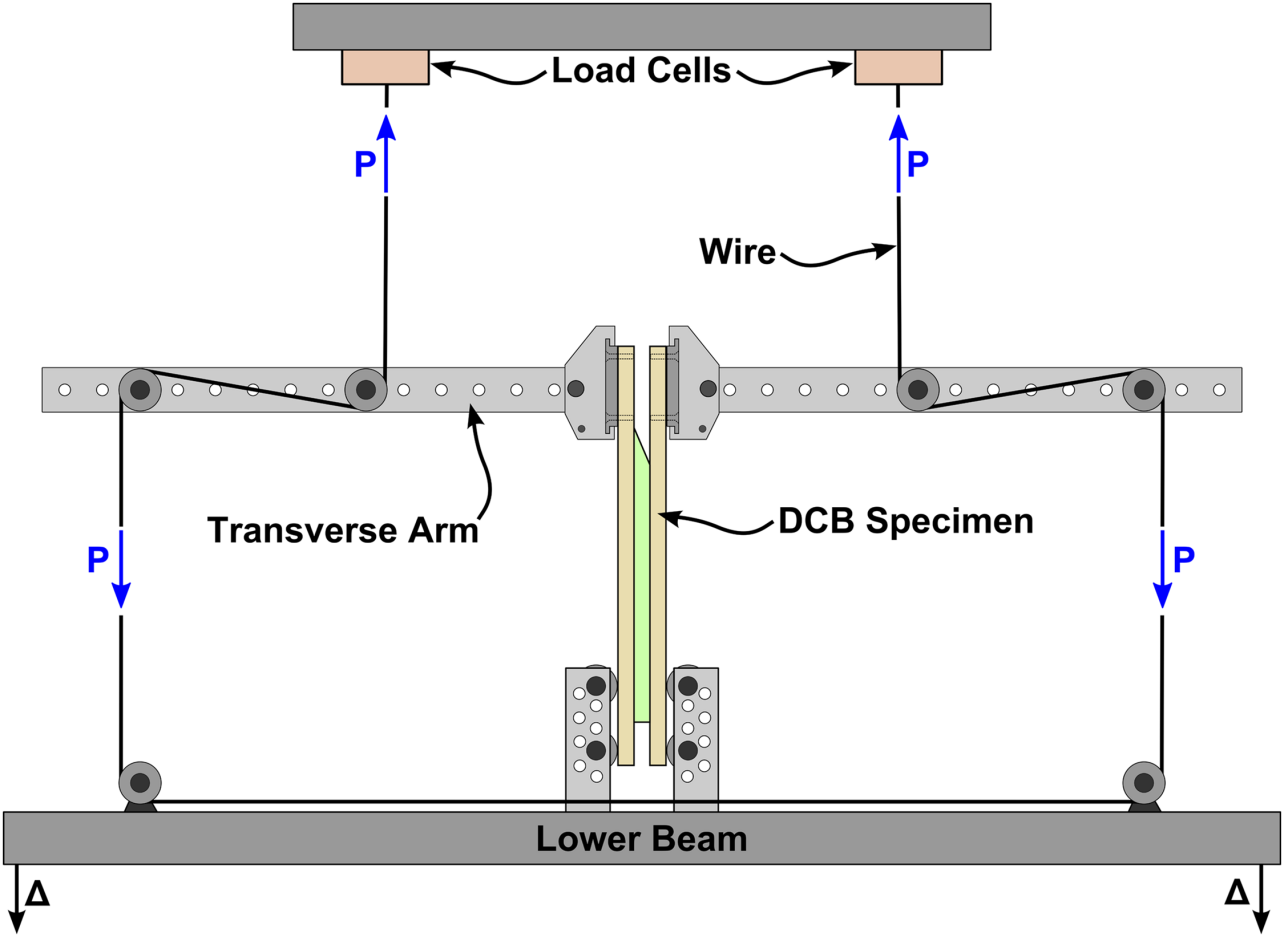
Schematic illustration of the double cantilever beam test set-up.

To correctly evaluate the different stages in the FBG response, a stable and controlled crack growth is required. However, the standard test methods used to characterise the macroscale fracture energy provide an unstable crack growth, particularly in Mode II loading. To overcome this, the fracture test machine developed by Sørensen [29], shown in Fig 20, was used. In this testing apparatus, the loading is applied through moments, providing a stable crack growth in the range of mode I to Mode II. Moreover, this testing apparatus allows the test to be stopped without decreasing the applied load, making it possible to perform measurements in a process that simulates continuous crack growth conditions.

To perform the loading, wires apply an equal transverse force to the transverse arms, which are attached to the DCB beams. The position where the wires are connected to the transverse arms defines the moments applied to each DCB beam, meaning that different wire positions will provide different loading types, from pure Mode I to pure Mode II. The force in the wires is measured by two load cells. One extensometer and two LVDTs (linear variable differential transformers) measure the crack face opening and sliding in the DCB specimen.

#### DCB Specimen Manufacturing

Two plates with dimensions of 700 × 1000 mm and a thickness of approximately 7 mm were produced using multiaxial glass fibre. Ten layers of fabric per plate were used, consisting of two triaxial fabrics (Saertex Triax S32E4590) as skin layers and eight unidirectional central layers (Saertex S35EU910). The layup stacking of the laminates was [90/ + 45/ − 45/0_4_/0_4_/ + 45/ − 45/90], and the backing of the unidirectional layers was facing outwards, away from the central plane. The plates were made by hand lay-up of dry fibre fabric, followed by epoxy impregnation (Momentive-Epikote/Epikure-100:30) by vacuum infusion at 50°C for 5 hours and post-curing at 80°C for 3 hours. The plates were glued using a commercial structural adhesive (Momentive-Epikote/Epikure MGS BPR 135G/137G), and 7 mm spacers were used to obtain a well-defined specimen thickness and geometry. A thin slip foil was placed on the edge of the structural adhesive to act as a pre-crack and ease crack initiation.

An array of 5 uncoated single-mode FBG sensors (5 gratings in one optical fibre), with a length of 10 mm, were embedded in the interface of the laminate plate with the structural adhesive. The gratings in the array were spaced 10 *mm* from each other, and the first grating was positioned 10 *mm* from the edge of the adhesive.

**Fig 21:**
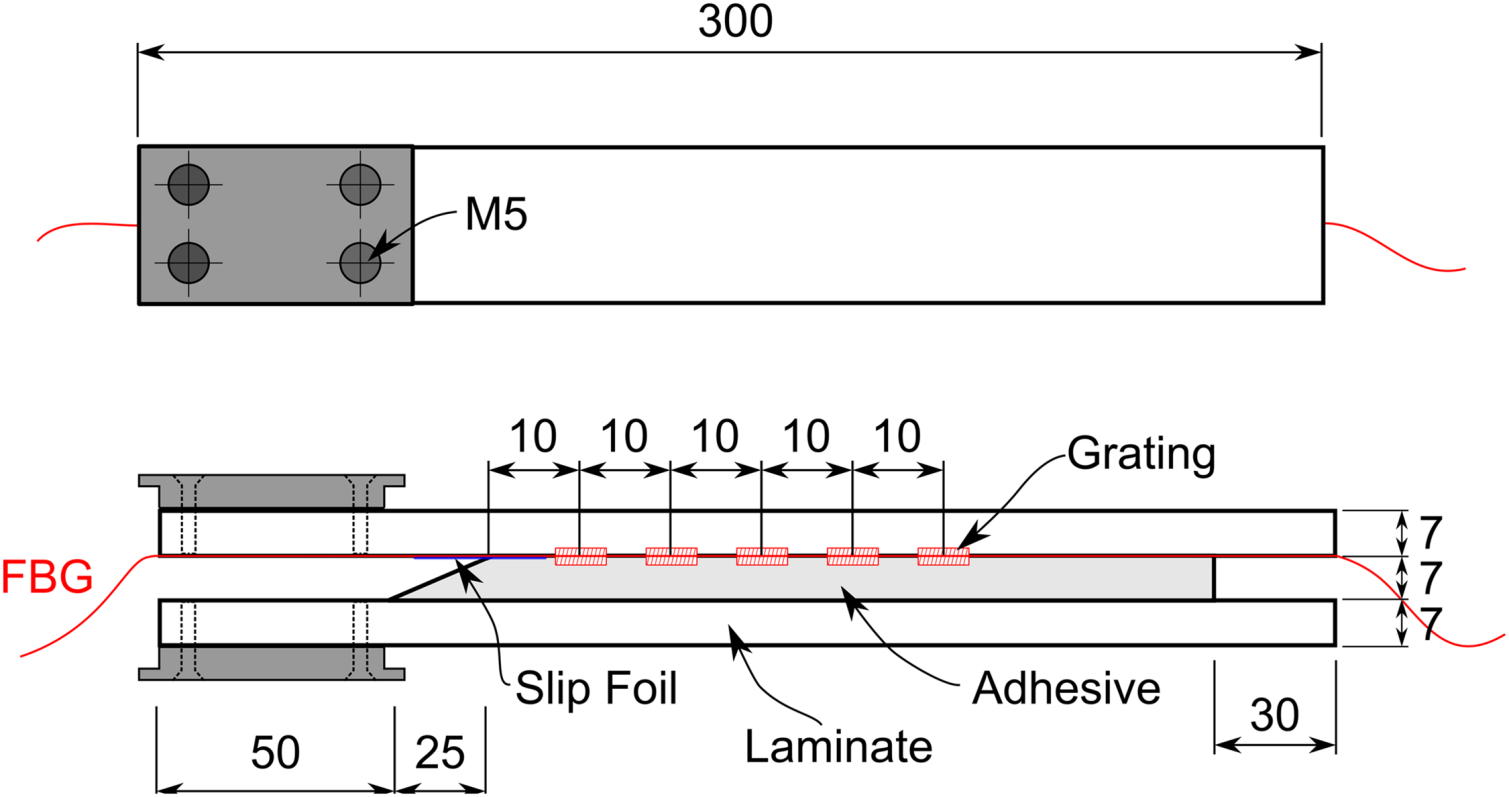
Sketch of the specimen geometry and FBG sensor position.

Five specimens, 30 mm in width, were cut from the sandwich plates. Steel parts were fixed to each beam by 4 steel screws (M5) by an epoxy adhesive (Scotch-Weld DP 460 from 3M, hardened at 40°C for two hours). The DCB dimensions, different components, and fibre grating locations are shown in Fig 21.

### Measurement Technology

#### Digital Image Correlation Technique

The digital image correlation (DIC) technique was used during the DCB fracture testing to determine the presence of specific phenomena caused by the crack, such as non-uniform strain or transverse stress, and correlate it with the FBG sensor output. The DIC technique is a non-contact optical method that can correlate the deformation/strain in a material by tracking changes in a random pattern on the specimen.

**Fig 22:**
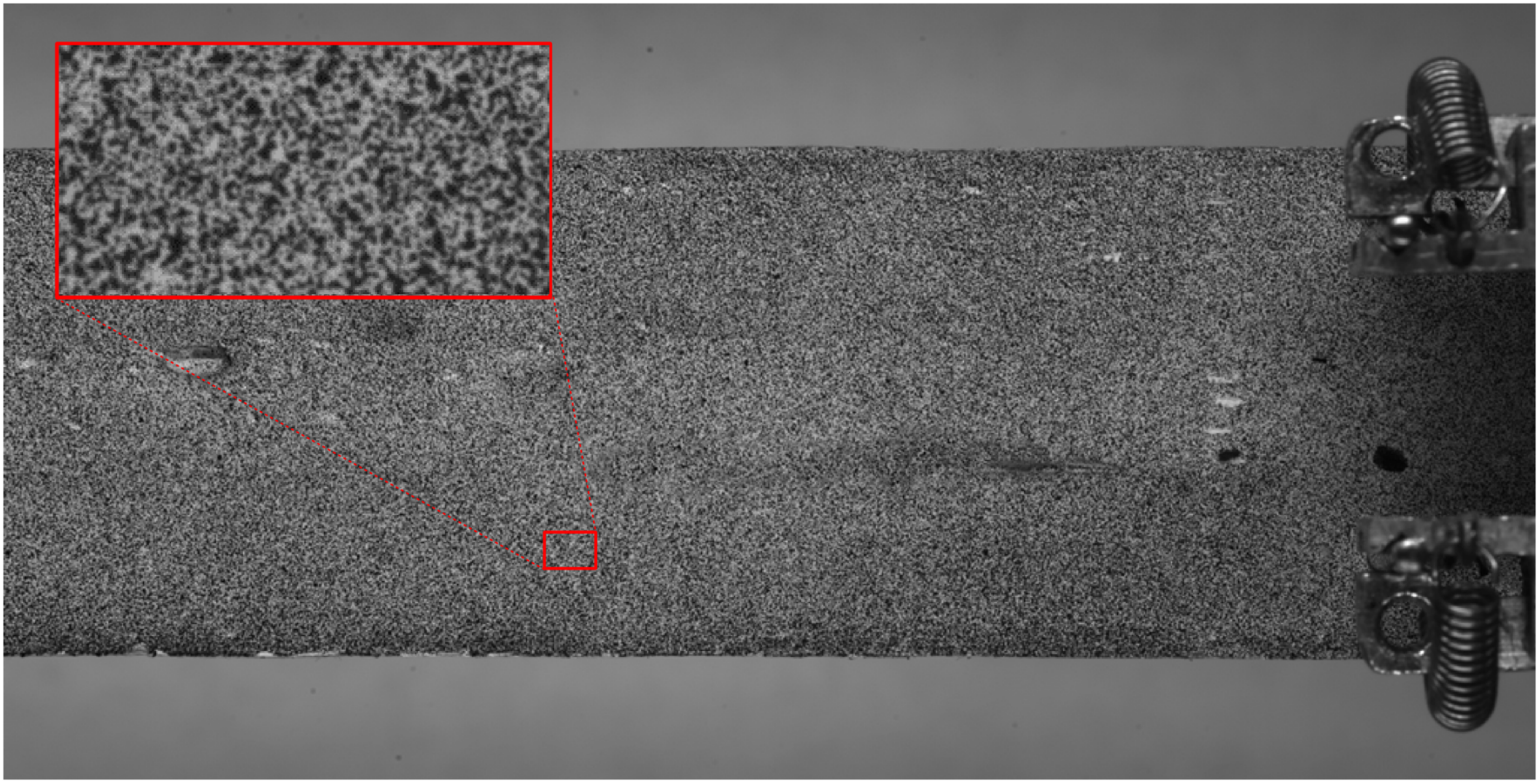
DIC pattern painted on the side surface of the DCB specimen.

A pattern was painted on the side surface of the DCB specimen, as shown in Fig 22, and ARAMIS V6.3 software was used to calculate the strains in each measurement. To perform the measurements, ARAMIS recognises the surface pattern in the unloaded specimen and allocates coordinates to the image pixels. Then, ARAMIS compares the pattern in the loaded specimen picture and, by tracking the changes, calculates the displacement and consequently the strain distribution in the specimen face. The facet parameters used for strain calculation were 60 × 60 pixel facets with a facet step of 15 pixels, which corresponds to a 45 pixel overlapping area [30].

#### Fibre Bragg Grating Optical Spectral Analyser System

**Fig 23:**
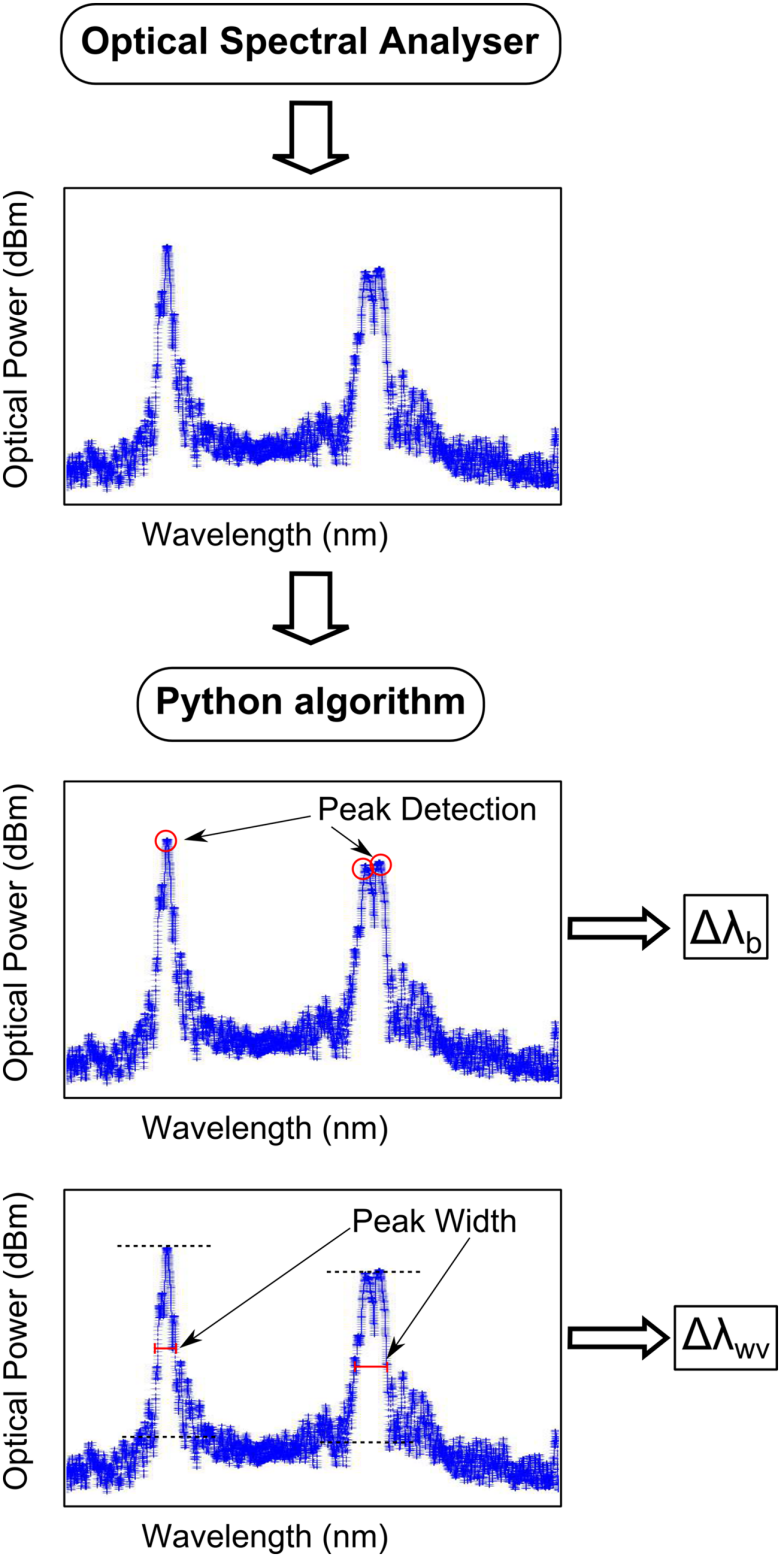
Algorithm for calculating the wavelength shift Δ*λ*_*b*_ and the width variation of the reflected peak Δ*λ*_*WV*_ from the reflected optical spectrum.

The FBG sensor was connected to an optical spectral analyser (OSA) *FS2200- Industrial BraggMeter* from FiberSensing^TM^ [31]. Each measurement performed by the OSA is a file with 20000 points, corresponding to the reflected light spectrum for the bandwidth from 1500 to 1600 *nm*. To manage this amount of data, an algorithm using *Python* was developed that computes from the reflected spectrum the wavelength shift Δ*λ*_*b*_ and the increase in width of the reflected peak Δ*λ*_*WV*_. Similar to the DIC technique, the algorithm uses the first reflected optical spectrum, measured in the unloaded specimen, to calculate the variation in the wavelength shift and increase in reflected peak width for each measurement (see Fig 23).

To calculate the wavelength shift Δ*λ*_*b*_, the algorithm detects the maximum reflected optical power of each grating and then computes Δ*λ*_*b*_ in relation to the original reflected peak. If the reflected peak is distorted or shows a split shape, the algorithm interpolates Δ*λ*_*b*_ between the maximum points in the grating bandwidth and the last maximum peak before the split occurred. To calculate the width of the reflected peak *λ*_*WV*_, the algorithm determines the maximum and minimum reflected optical power for each grating and measures the peak width at half maximum optical power ((*maximum* + *minimal*)/2). It then computes the width variation of the reflected peak Δ*λ*_*WV*_ relative to the original reflected peak width.

## Experimental Results and Discussion

**Fig 24:**
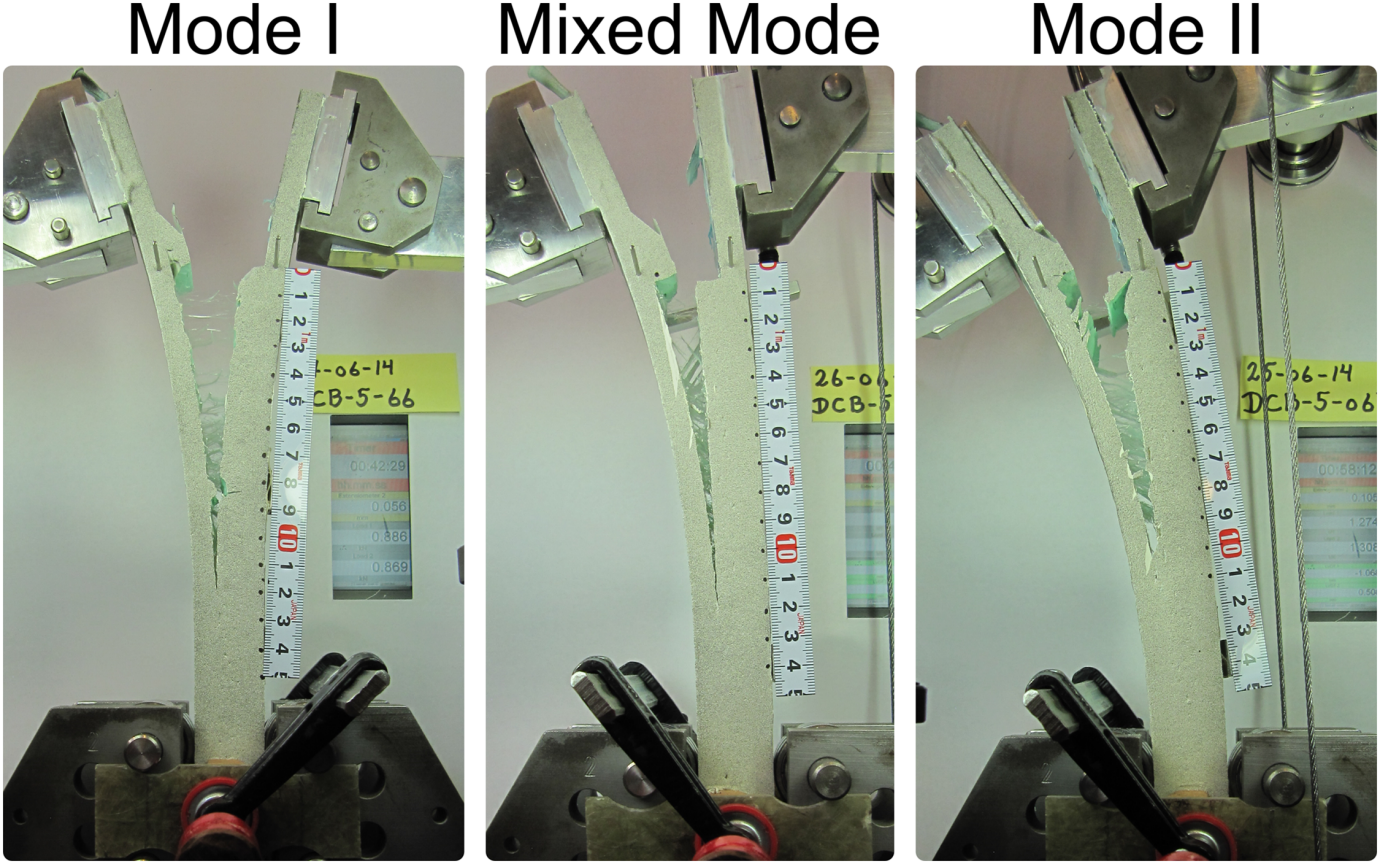
Fracture modes addressed in the DCB testing.

Three DCB specimens loaded with different fracture modes, namely, Mode I (*ψ*_*nom*_ = 0°), Mode II (*ψ*_*nom*_ = 90°), and Mixed Mode (*ψ*_*nom*_ = 68°), are shown in Fig 24. The type of fracture mode performed for each DCB specimen is described in Table 4.

**Table 4:** Fracture Modes Tested.

DCB specimen	1	2	3	4	5
**Fracture Mode**	I/II	I	I	I	II
	I				I/II
	II				I

In specimens 1 and 5, an initial fracture test was performed until the crack reached the middle of the FBG array. Then, the test was restarted with a different fracture mode to simulate a change in the loading conditions and evaluate the ability of the sensors to measure a crack independent of the loading configuration.

**Fig 25:**
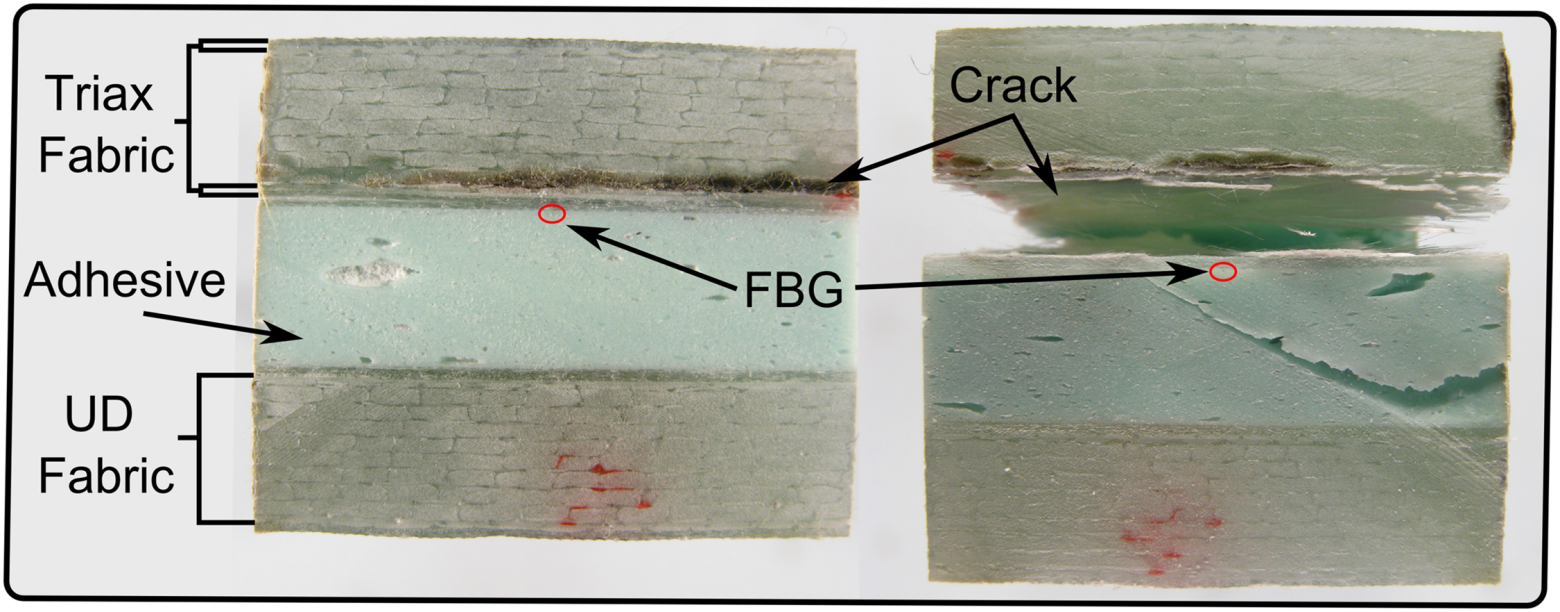
Crack face in the DCB specimen.

A critical issue found when using FBG sensors embedded in the FRP material is the possibility of damaging the sensor. If the crack changes it path direction and crosses the optical fibre, this will cut the signal, losing all the gratings ahead of that point. To avoid this situation, crack initiation between the triaxial laminate and the unidirectional laminate was promoted by using a thin slip foil, as shown in Fig 25. Thus, the FBG sensor was *0.3–0.4 mm* away from the crack face, protected from any damage, as long as the crack did not change direction. As planned, the crack followed the predicted path for the Mode II and mixed mode testing. However, the crack did change direction during the test of specimens 2, 3 and 4 under Mode I, losing some of the gratings during the propagation of the crack. Nevertheless, sufficient data was acquired during these tests, allowing the validation of the structure-material-sensor model in mode I fracture.

**Fig 26:**
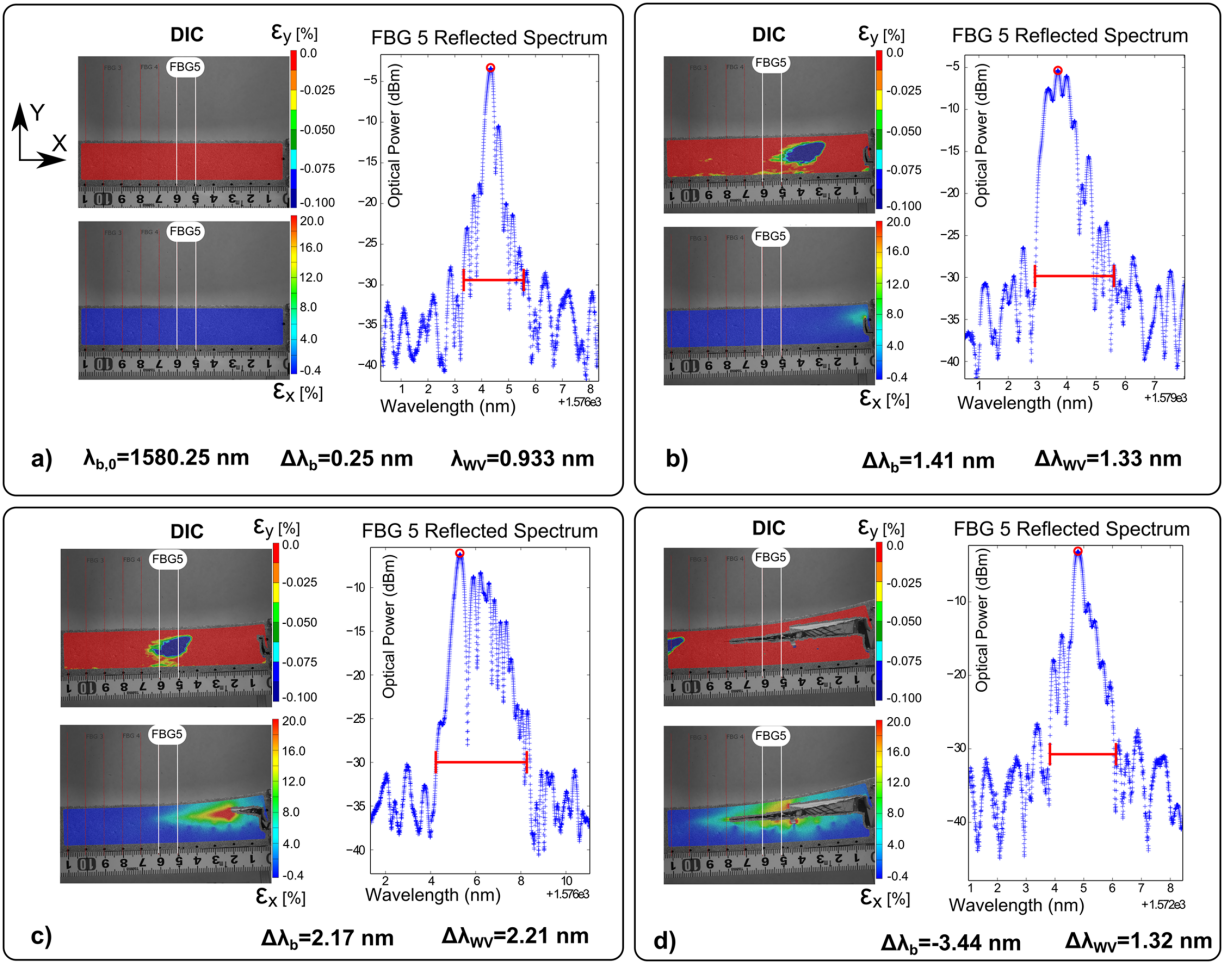
FBG sensor output during crack growth in Mode II. a) Before crack initiation; b) crack growth: compression field at grating position; c) crack growth: non-uniform strain at grating position; and d) crack growth and passing all grating length.

The FBG response and DIC strain measurements during crack growth in a DCB specimen are shown in Fig 26. The reflected peak of the Bragg grating that is situated closer to the adhesive edge corresponds to FBG 5, with an original reflected peak of *λ*_*b*_ = 1580 *nm*. All the different crack features/phenomena that can change the shape of the reflected peak were identified and correlated with a specific FBG response. The left row pictures are DIC measurements, where the top shows the negative component of strain in the *y* direction, *ɛ*_*y*_ (“compression” strain), and the bottom shows the strain in the *x* direction, *ɛ*_*x*_.

The blue spot in the top DIC measurements is the compression field *ɛ*_*y*_ formed ahead of the crack tip. The colour gradient in the bottom DIC measurements is the indication of longitudinal variation of strain that moves with the crack tip.

By analysing the three figures, it is possible to identify all the different stages in the sensor response during crack growth, as described previously. In Fig 26a), before the crack reaches the proximity of the grating, the material accumulates uniform strain. This induces a uniform wavelength shift in the sensor response, from *λ*_*b*,0_ = 1580.00 *nm* to *λ*_*b*_ = 1580.25 *nm*. Next, the compression field formed ahead of the crack tip reaches the grating area. This modified the shape of the reflected peak to a split peak shape, with a peak width increase of Δ*λ*_*WV*_ = 1.33 *nm*, as shown in Fig 26b). Additionally, an increase in the wavelength shift, Δ*λ*_*b*_ = 1.41 *nm*, was measured, which was caused by the loading increase that consequently increased the strain in the specimen. If the crack continues to grow, the grating will gradually experience the influence of the crack singularity (region dominated by stress concentration), which creates a non-uniform strain distribution around the sensor length. This non-uniform strain will create a change in the reflected peak shape, where multiple reflected peaks appear and the peak width increases Δ*λ*_*WV*_ = 2.21 *nm*, as shown in Fig 26c). Following the previous stages, an increase in the wavelength shift was measured, Δ*λ*_*b*_ = 2.17 *nm*, which was caused by the continuous load increase. Finally, after the crack passed the grating full length, the reflected peak width decreased, Δ*λ*_*WV*_ = 1.32 *nm*, and the shape of the reflected peak gradually recovered its original shape, as shown in Fig 26d). However, the wavelength shift continued to vary, Δ*λ*_*WV*_ = −3.44 *nm*, following the increase of load and strain in the specimen.

Due to the large quantity of data saved during the fracture tests, it is impossible to present all the results in this article. However, to provide the reader with a better understanding of the crack detection technique, three movies from the three fracture modes tested (S1, S2 and S3 Videos) are presented in the *Supporting Information*. In each movie is shown the reflected spectrum from the FBG array, a picture of the specimen during the test, and the DIC results, synchronised with the wavelength shift Δ*λ*_*b*_ and peak width variation Δ*λ*_*WV*_ measured during the test.

**Fig 27:**
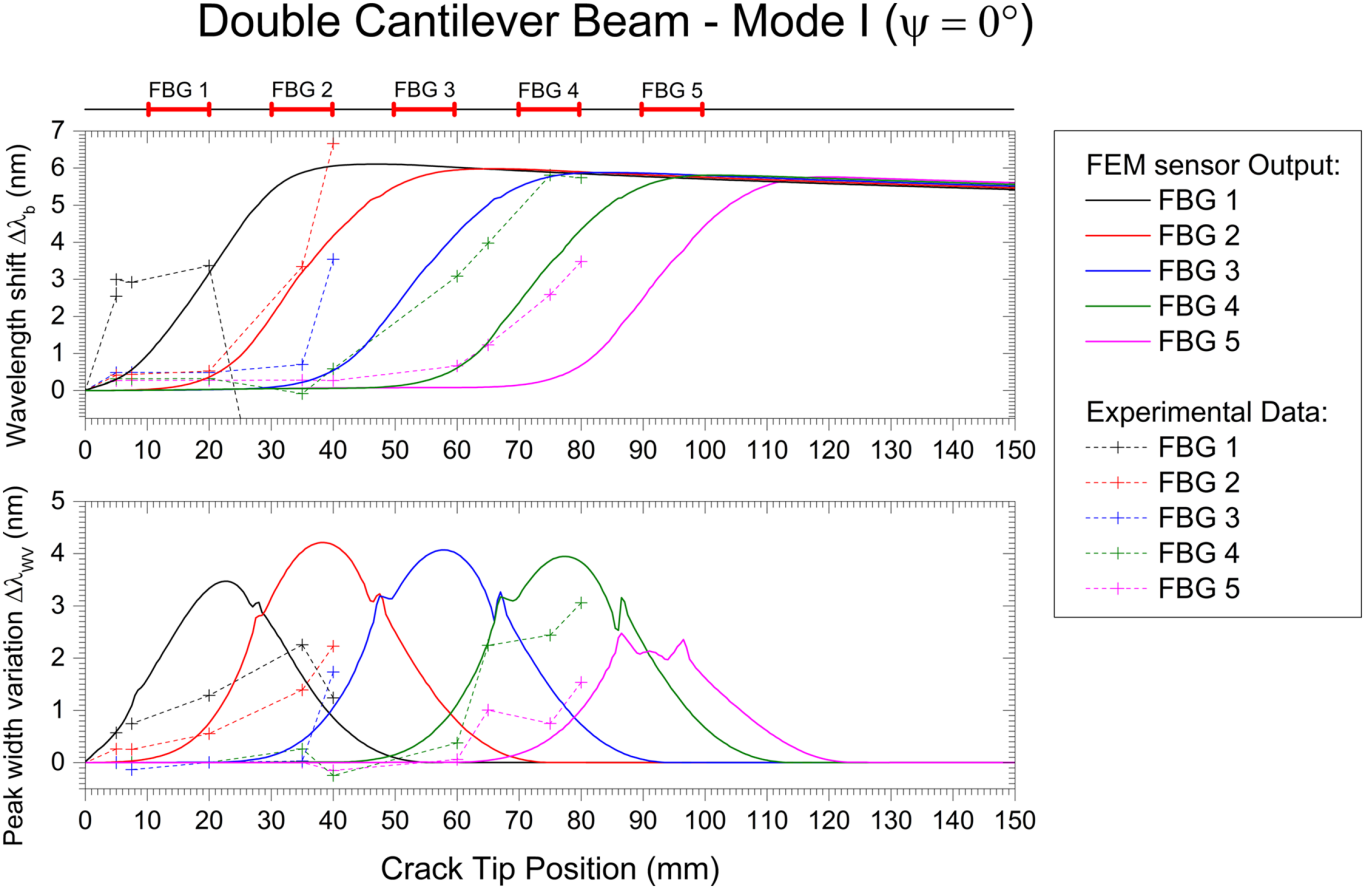
Embedded FBG sensor output in a DCB specimen under Mode I fracture testing: numerical and experimental results.

**Fig 28:**
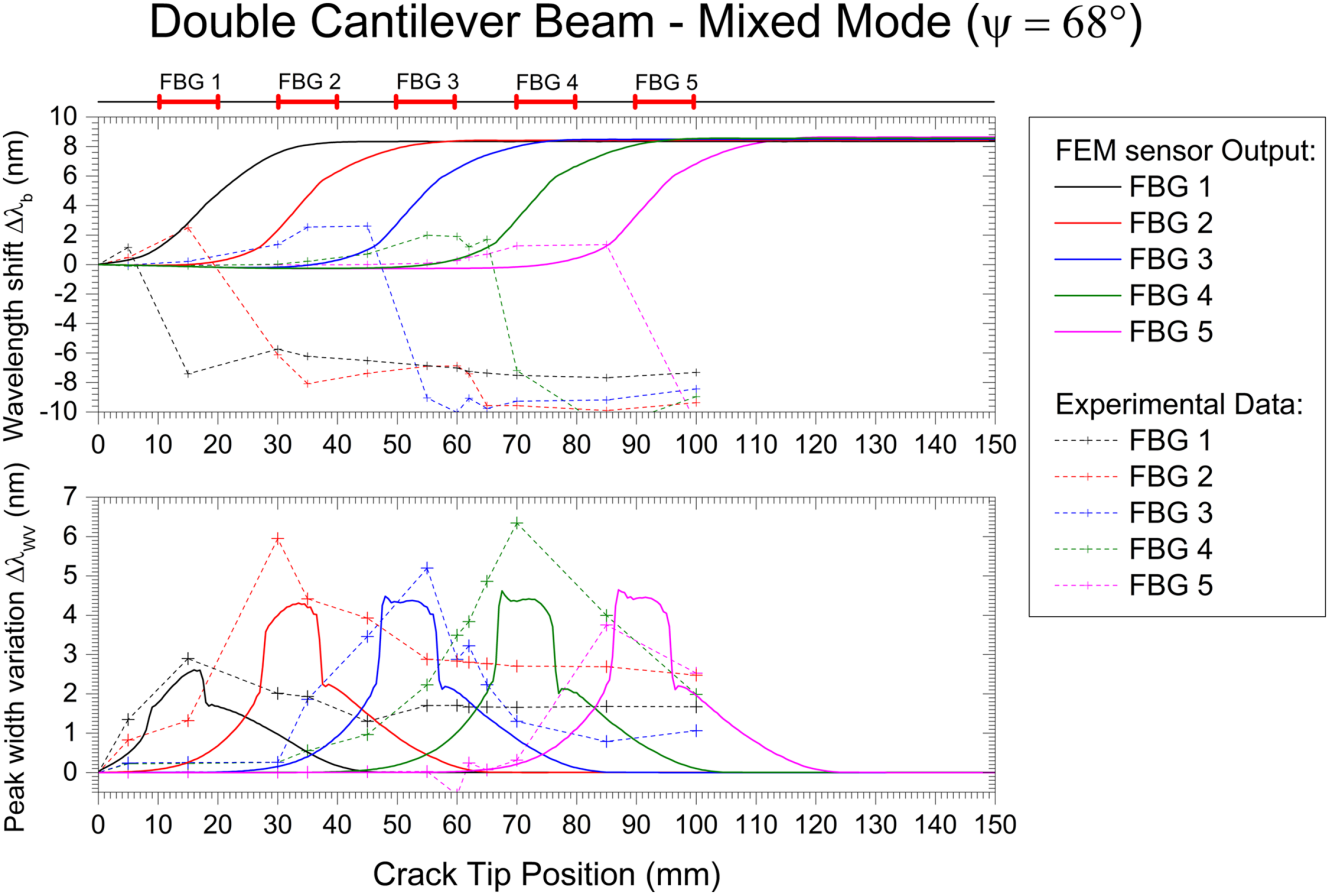
Embedded FBG sensor output in a DCB specimen under Mixed Mode fracture testing: numerical and experimental results.

**Fig 29:**
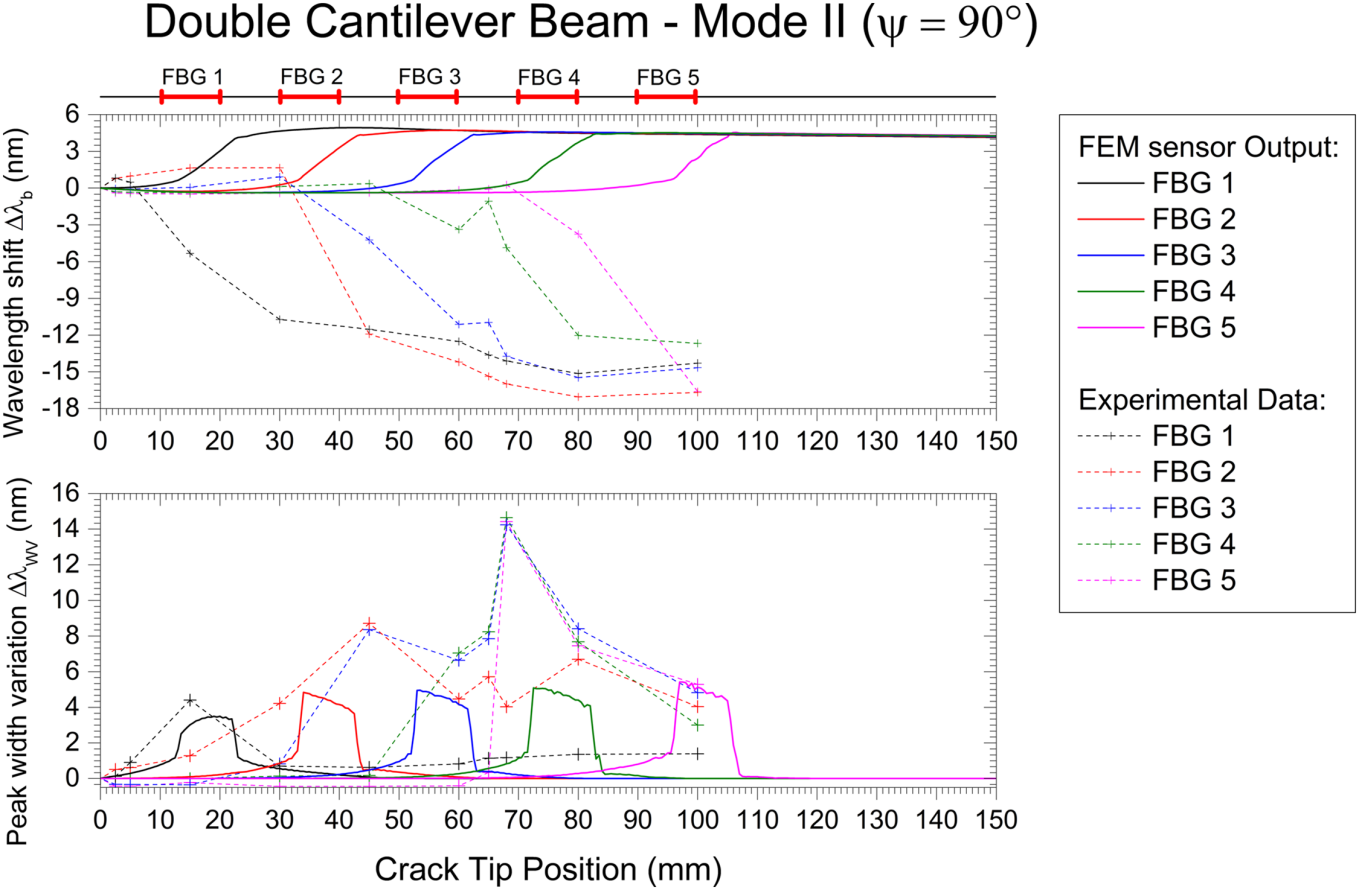
Embedded FBG sensor output in a DCB specimen under Mode II fracture testing: numerical and experimental results.

The Mode I, Mode II and Mixed Mode fracture testing experimental results are compared with the numerical simulation in Figs 27, 28 and 29. The wavelength shift Δ*λ* and width variation of the reflected peak Δ*λ*_*WV*_ were computed from the measured reflected spectrum given by the OSA of the Braggmeter using the developed algorithm. The crack tip position was calculated using the DIC technique.

Note that the goal of this technique is to detect cracks, not to quantify stress or strain. Thus, the magnitude of the measured values can vary, but the information obtained that is used to determine the presence of the crack is accurate. With this, a good agreement between the experiments and simulation was found.

The wavelength shift, Δ*λ*_*b*_, difference between the experimental results and the numerical prediction is due to the loading and geometric dependency of this parameter; i.e., small variations in the position of the sensor or a different crack growing path can vary the measured strain. For the Mode II and Mixed Mode cases, the path of the crack shifts during the test, changing the position of the grating from the top crack face to the bottom face, as shown in Fig 25. This causes a change in the measured strain, Δ*λ*_*b*_), from positive to negative. However, in terms of absolute values, both cases exhibited the predicted behaviour.

As previously discussed, the main advantage of this monitoring technique is the use of two different FBG output parameters, Δ*λ*_*b*_ and Δ*λ*_*WV*_, to determine the presence of the crack and to track its growth. The wavelength shift, Δ*λ*_*b*_, is a parameter related to the strain level in the structure, but it is dependent on the loading and geometry configuration. This can be observed in mode I loading, where an increase of Δ*λ*_*b*_ is observed, and in Mode II/Mixed, where a decrease in Δ*λ*_*b*_ occurs. However, the rapid increase in the magnitude of Δ*λ*_*b*_ is caused by a damage event that reduces the stiffness of the structure. In contrast, the width variation of the reflected peak, Δ*λ*_*WV*_, is a parameter that only depends on the presence of a crack, independent of geometry and loading type. The width of the reflected peak, Δ*λ*_*WV*_, increases when the crack is near the grating area, being low in magnitude before and after the crack passes. In summary, these two parameters are a good indicator of the presence of cracks, and a structural health monitoring system based on FBG sensor technology needs to evaluate both variables to accurately detect such damage.

As an example of this crack detection methodology, refer to Fig 28, when the crack tip is at 50 mm (beginning of FBG3). The value of Δ*λ*_*b*_ is larger for FBG1 and FBG2; the FBG3 Δ*λ*_*b*_ value is starting to increase; and the FBG4 and FBG5 Δ*λ*_*b*_ values are still low. This indicates that the compliance of the material is changing in location 3. However, the value of Δ*λ*_*WV*_ is higher for FBG3 and FBG4, and it is lower for FBG1, FBG2, and FBG5. This result indicates that these two locations, 3 and 4, are experiencing specific fracture features ahead of the crack tip (compression and non-uniform strain). Using this information, we can confidently predict the crack position, which has already passed positions 1 and 2 and is located at position 3.

## Summary and Conclusions

Inspired by the change in the “conventional” structure design philosophy to a damage tolerant structural design, through the use of damage tolerant materials combined with structural health monitoring techniques, an approach to detect damage in structures composed of composite materials and structural adhesive was outlined in this paper. This concept will eventually lead to a condition monitoring-maintenance, which consists of the detection of damage by sensors, characterisation of damage (type and size), and model predictions of residual life that will enable decision-making with respect to whether a structure should be repaired or replaced.

The ability of fibre Bragg gratings embedded in composite materials to detect and track cracks/delamination by identifying the response of a sensor to a specific fracture/damage phenomena was demonstrated. Three different mechanisms that can change the sensor output, namely, longitudinal strain *ɛ*_*xx*_, transversal stress *σ*_*y*, *z*_ and non-uniform strain *ɛ*_*xx*_(*x*), were described and linked with the different damage mechanisms that occur during a crack growth event. These different measurement concepts were incorporated into a finite element model of a delamination of a double cantilever beam to simulate the sensor output under different conditions. Using this technique, it becomes possible to extract information from the sensor output that is independent of the loading type, structure geometry and boundary conditions, depending only on the proximity of the crack and the material properties.

The *material-structure-sensor* model can be used as a design tool for applying this monitoring technology in different composite material structures, predicting the sensor output, and determining the optimised sensor-structure configuration. As per the authors’ vision, this *material-structure-sensor* model concept will make it possible to design structures in composite materials that can operate safely, even when in damaged conditions.

## Supporting Information

S1 File *Python* script to calculate the FBG sensor response from the FEM model. (PDF)

S1 Video Crack Monitoring in DCB under Mode I fracture. This movie shows the application of crack monitoring technique using FBG sensors in a DCB specimen under Mode I fracture. It is showed the reflected spectrum from the FBG array, a picture of the specimen during the test, and the DIC results, synchronized with the wavelength shift Δ*λ*_*b*_ and peak width variation Δ*λ*_*WV*_ results computed during the test. The movie is divided in two parts: in the first part the DIC technique shows the negative component of the strain field in the *y* direction, *ɛ*_*y*_; in the second part the DIC technique shows the strain field in the *x* direction, *ɛ*_*x*_.
(MP4)

S2 Video Crack Monitoring in DCB under Mixed Mode fracture. This movie shows the application of crack monitoring technique using FBG sensors in a DCB specimen under Mixed Mode fracture. It is showed the reflected spectrum from the FBG array, a picture of the specimen during the test, and the DIC results, synchronized with the wavelength shift Δ*λ*_*b*_ and peak width variation Δ*λ*_*WV*_ results computed during the test. The movie is divided in two parts: in the first part the DIC technique shows the negative component of the strain field in the *y* direction, *ɛ*_*y*_; in the second part the DIC technique shows the strain field in the *x* direction, *ɛ*_*x*_.
(MP4)

S3 Video Crack Monitoring in DCB under Mode II fracture. This movie shows the application of crack monitoring technique using FBG sensors in a DCB specimen under Mode II fracture. It is showed the reflected spectrum from the FBG array, a picture of the specimen during the test, and the DIC results, synchronized with the wavelength shift Δ*λ*_*b*_ and peak width variation Δ*λ*_*WV*_ results computed during the test. The movie is divided in two parts: in the first part the DIC technique shows the negative component of the strain field in the *y* direction, *ɛ*_*y*_; in the second part the DIC technique shows the strain field in the *x* direction, *ɛ*_*x*_.
(MP4)
