# Supplementary material for: Crack Detection in Fibre Reinforced Plastic Structures Using Embedded Fibre Bragg Grating Sensors: Theory, Model Development and Experimental Validation
Source: PLoS One. 2015 Oct 29;10(10):e0141495. doi: 10.1371/journal.pone.0141495 (PMC4626382; doi:10.1371/journal.pone.0141495)
Supplement: S1 File — (PDF) [file pone.0141495.s001.pdf]

S1 python script. *Python* script to calculate the FBG sensor response from the FEM model.

```
"""
Created by Gilmar Pereira---Contact email: gfpe@dtu.dk

This code is used as Abaqus Post-processing tool to predict the
FBG sensor output during crack Growth for a 2D Case.
"""
#Packages
import numpy as np
import scipy as sp
import sympy
import matplotlib.pyplot as plt
import math

"""
The code starts by loading the report files given by Abaqus.
Export 2 different files(sigma_22 and epsilon_11), using the path tool,
along the fiber optic position. This script will work for a 2D case.
For a 3D case, add a third file with the stress in the transverse
direction (sigma_33).
"""
#Load file sigma_22
filess22='filename_s22.txt'
#Load file epsilon_11
fileLE11='filename_E11.txt'
"""Script Input"""
#Size of elements (mm)
se=0.5
#Length of the grating(mm)
lg=10
#Number of gratings
ng=5
#Space between gratings(mm)
sbg=10
#Number of elements per grating
neg=lg*se

"""Optical Fiber input Parameters"""
#Original wavelength
LFBG=[1528.813*10**(-9),1541.317*10**(-9),1554.252*10**(-9),
1567.121*10**(-9),1580.246*10**(-9)]
#PhotoElastic Parameter
pe=0.215
#Initial refractive index
n0=1.45
#Optical fiber young's module
Ef=75*10**3
#Poisson's coefficient of the optical fiber
vf=0.17
#Directional refractive index parameters.
p11=0.121
p12=0.270
# Inital Grating period calculation
igp=[x/(2*n0) for x in LFBG]
```

```
"""-------------------------------------------------------------
Loading Data: FBGX-Y; x- is the FBG number,
Y is the element number- starting from 0 to neg."""
names =('x',)
formats=('f8',)
for x in np.arange(ng,0,-1):
    FBGref= 'FBG'+str(x)
    for i in np.arange(neg,0,-1):
        names= names + (FBGref+'-'+str(i),)
        formats=formats+ ('f8',)
dtypes = {'names' : names,'formats': formats}
#Loading file
s22=np.loadtxt(filess22, dtype=dtypes,skiprows=5)
LE11=np.loadtxt(fileLE11, dtype=dtypes,skiprows=5)
"""-------------------------------------------------------------
Calculation of the Wavelength shift - uniform strain contribution,
wlv: wavelength variation: average of the strain in the length
of the grating """
LEaverage={}
for b in range(1,ng+1):
    LEaverage['FBG'+str(b)]= []
for a in range(0,size(LE11)):
    for f in range(1,ng+1):
        temp=0
        temp2=0
        FBGname= 'FBG'+str(f)
        for l in range(1,neg+1):
            sensorname=FBGname+'-'+str(l)
            temp=temp+ LE11[sensorname][a]
        temp2=temp/neg
        LEaverage[FBGname].append(temp2)
# Wavelength shift calculation (Units of wavelength in nm)
WaveShift={}
for b in range(1,ng+1):
    Fbgname= 'FBG'+str(b)
    WaveShift[Fbgname]=[x*LFBG[b-1]*(1-pe)*10**9 for x in
                        LEaverage[Fbgname]]
"""-------------------------------------------------------------
Reflected Peak Width Variation calculation: Transversal Stress
Using an average of the transverse stress values. """
s22average={}
for b in range(1,ng+1):
    s22average['FBG'+str(b)]= []
for a in range(0,size(s22)):
    for f in range(1,ng+1):
        temp=0
        temp2=0
        FBGname= 'FBG'+str(f)
        for l in range(1,neg+1):
            sensorname=FBGname+'-'+str(l)
            temp=temp+ s22[sensorname][a]
        temp2=temp/neg
        s22average[FBGname].append(temp2)
# Peak Width Variation calculation
#Equation fixed component
fce=(((1+vf)*p12-(1+vf)*p11)*n0**3)/Ef
```

```
Wavegap={}
for b in range(1,ng+1):
    Fbgname= 'FBG'+str(b)
    #Units of the wavelength in nm (*10**9)
    Wavegap[Fbgname]=[abs(abs(x)*fce*igp[b-1]*10**9) for x in
                                s22average[Fbgname]]
"""-------------------------------------------------------------
Reflected Peak Width Variation calculation: Non-uniform strain
Using maximum and minimum strain along the grating. """
graperiodmax={}
graperiodmin={}
Wavegap2={}
for b in range(1,ng+1):
    graperiodmax['FBG'+str(b)]= []
    graperiodmin['FBG'+str(b)]= []
    Wavegap2['FBG'+str(b)]= []
for a in range(0,size(LE11)):
    for f in range(1,ng+1):
        maxim= None
        minm= None
        FBGname= 'FBG'+str(f)
        for l in range(1,neg+1):
            sensorname=FBGname+'-'+str(l)
            if LE11[sensorname][a]>maxim or maxim== None:
                maxim=LE11[sensorname][a]
            if LE11[sensorname][a]<minm or minm== None:
                minm=LE11[sensorname][a]
        graperiodmax[FBGname].append(igp[f-1]*(1+(1-pe)*maxim))
        graperiodmin[FBGname].append(igp[f-1]*(1+(1-pe)*minm))
        #Units of wavelength in nm (*10**9)
        Wavegap2[FBGname].append(2*n0*(graperiodmax[FBGname][a]
                -graperiodmin[FBGname][a])*10**9)
"""-------------------------------------------------------------
Writing file with Results """
file = open("Name_of_the_file.txt", "w")
file.write("Step increment\t")
for b in range(1,ng+1):
    file.write("Wavelength Shift (nm)- FBG" + str(b)+'\t')
for c in range(1,ng+1):
    file.write("Peak Splitting (nm)- FBG" + str(c)+'\t')
file.write('\n')

for a in range(0,size(LE11)):
    file.write('%5f \t' %(LE11['x'][a]))
    for b in range(1,ng+1):
        Fbgname= 'FBG'+str(b)
        file.write('%5f \t' %(WaveShift[Fbgname][a]))
    for b in range(1,ng+1):
        Fbgname= 'FBG'+str(b)
        file.write('%5f \t' %(Wavegap[Fbgname][a]
                +Wavegap2[Fbgname][a]))
    file.write('\n')
file.close()
```
